# Differential histone acetylation and super-enhancer regulation underlie melanoma cell dedifferentiation

**DOI:** 10.1172/jci.insight.166611

**Published:** 2024-02-06

**Authors:** Karen Mendelson, Tiphaine C. Martin, Christie B. Nguyen, Min Hsu, Jia Xu, Claudia Lang, Reinhard Dummer, Yvonne Saenger, Jane L. Messina, Vernon K. Sondak, Garrett Desman, Dan Hasson, Emily Bernstein, Ramon E. Parsons, Julide Tok Celebi

**Affiliations:** 1 Department of Dermatology, NYU Grossman School of Medicine, New York, New York, USA.; 2 Department of Oncological Sciences and; 3 Tisch Cancer Institute, Icahn School of Medicine at Mount Sinai, New York, New York, USA.; 4 Graduate School of Biological Sciences, Icahn School of Medicine, New York, New York, USA.; 5 Department of Dermatology, University Hospital of Zurich, Zurich, Switzerland.; 6 Department of Medicine, Columbia University Medical Center, New York, New York, USA.; 7 Department of Pathology and Cell Biology, USF Morsani College of Medicine, Tampa, Florida, USA.; 8 Moffitt Cancer Center, Tampa, Florida, USA.; 9 Department of Pathology, Molecular and Cell Based Medicine, Icahn School of Medicine at Mount Sinai, New York, New York, USA.; 10 Department of Pathology, NYU Grossman School of Medicine, New York, New York, USA.

**Keywords:** Dermatology, Melanoma

## Abstract

Dedifferentiation or phenotype switching refers to the transition from a proliferative to an invasive cellular state. We previously identified a 122-gene epigenetic gene signature that classifies primary melanomas as low versus high risk (denoted as Epgn1 or Epgn3). We found that the transcriptomes of the Epgn1 low-risk and Epgn3 high-risk cells are similar to the proliferative and invasive cellular states, respectively. These signatures were further validated in melanoma tumor samples. Examination of the chromatin landscape revealed differential H3K27 acetylation in the Epgn1 low-risk versus Epgn3 high-risk cell lines that corroborated with a differential super-enhancer and enhancer landscape. Melanocytic lineage genes (MITF, its targets and regulators) were associated with super-enhancers in the Epgn1 low-risk state, whereas invasiveness genes were linked with Epgn3 high-risk status. We identified the *ITGA3* gene as marked by a super-enhancer element in the Epgn3 invasive cells. Silencing of ITGA3 enhanced invasiveness in both in vitro and in vivo systems, suggesting it as a negative regulator of invasion. In conclusion, we define chromatin landscape changes associated with Epgn1/Epgn3 and phenotype switching during early steps of melanoma progression that regulate transcriptional reprogramming. This super-enhancer and enhancer-driven epigenetic regulatory mechanism resulting in major changes in the transcriptome could be important in future therapeutic targeting efforts.

## Abstract 2

Chromatin landscape changes associated with Epgn1/3 subtypes and phenotype switching during the early steps of melanoma progression regulate transcriptional reprogramming.

## Introduction

In melanoma, a specific dedifferentiation program referred to as phenotype switching is well recognized, where proliferating cells, due to tumor cell intrinsic and cell extrinsic cues, shift their transcriptomes and become slow cycling and invasive (1–3). A hallmark of the transcriptional programs regulating this process is the transcription factor (TF) microphthalmia-associated TF (MITF). MITF is a melanocytic lineage identity gene. In melanoma, it promotes cell proliferation, differentiation, and survival; regulates genes in the pigmentation pathway; and suppresses invasion and senescence (3). Dedifferentiation is associated with loss of MITF, its target genes and regulators, metastasis (4), intrinsic resistance to mitogen-activated protein kinase (MAPK) inhibitors (5), and immunotherapy resistance (6). In addition to MITF, SOX10 is another TF associated with proliferative cellular states, whereas AP1 and TEAD family members are associated with invasive cellular states (7, 8). High levels of AP1, EGFR, NGFR, and WNT are recognized as dedifferentiation markers (7, 9–11). Based on single-cell RNA-Seq, a 4-stage model has been described beyond the 2 states (proliferative and invasive); melanocytic, transitory, neural crest–like, and undifferentiated (12). Similarly, 2 main states (proliferative and invasive) and an intermediary status have also been proposed (13). While transcriptional reprogramming during dedifferentiation or phenotype switching is well recognized, mechanisms affecting the transcriptional output of the cell such as those involving the epigenome are largely uncharacterized. Additionally, reprogramming that occurs in the early stages of melanoma progression is less studied. The majority of the studies have been in the metastatic setting.

We have previously reported an epigenetic gene signature (122-epigenetic genes) that classifies primary cutaneous melanomas into 2 major categories; low- (Epgn1) and high-risk (Epgn3) subsets (14). These data suggest that epigenome deregulation could underlie this process. As we dissected the epigenetic gene groups, we uncovered that Epgn1 and Epgn3 subsets correlate with proliferative and invasive cellular states of phenotype switching, respectively. Here, we investigated the chromatin landscape changes and epigenetic mechanisms underlying these states.

## Results

### An epigenetic gene signature (Epgn1/Epgn3) underlies melanoma cell dedifferentiation (or phenotype switching).

**Figure 1:**
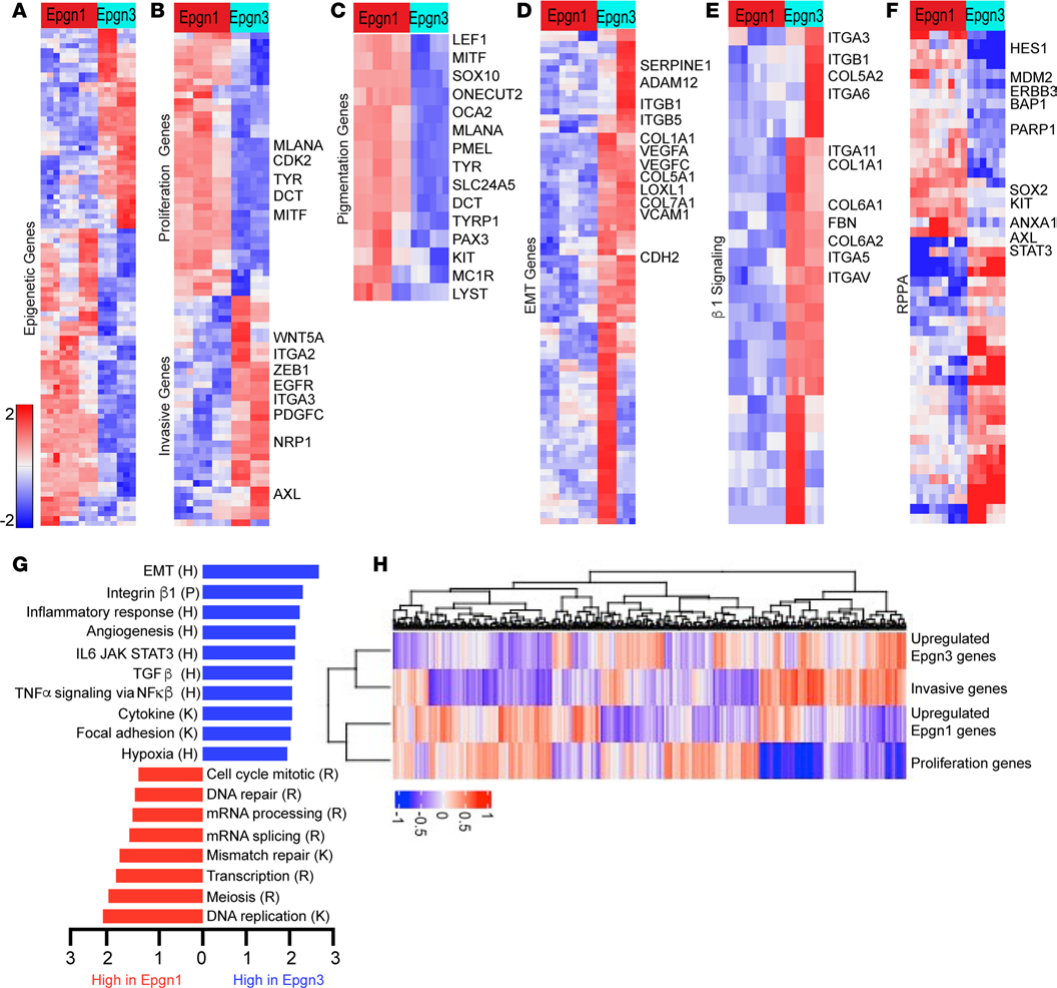
Epigenetic gene signature classifier (Epgn1/Epgn3) underlies reprogramming of melanoma cells from a proliferative to an invasive cellular state. RNA-Seq of Epgn1 cell lines (*n* = 3; WM35, YUPEET, WM983A) and Epgn3 cell lines (*n* = 2; WM1552C, YUCHIME). The top bar indicates cellular subtypes (Epgn1 [red] and Epgn3 [light blue]) as characterized by the 122-epigenetic signature (Supplemental Figure 1A). Each row of the heatmap indicates a differentially expressed gene, and each column represents a *BRAFV600* mutant cell line (*n* = 5; each in triplicate). Differentially expressed genes are significant if *q* < 0.05 by Benjamini-Hochberg procedure and a linear fold-change ± 1.5. The heatmaps are color-coded on the basis of *Z* scores. (**A**) Supervised hierarchical clustering of 122 epigenetic genes and identification of Epgn1 and Epgn3 groups. (**B**) RNA-Seq analysis identifies increased expression of proliferative and differentiation genes (*MLANA, TYR, DCT, MITF*) in the Epgn1 group and increased expression of invasive genes (*WNT5A, ITGA2, ZEB1, EGFR, ITGA3, PDGFC, NRP, AXL*) in the Epgn3 group using the Hoek proliferative and invasive gene signature (2). (**C**) Pigmentation pathway genes (*MITF, MLANA, PMEL, TYR, DCT, TYRP1*) were uniformly upregulated in Epgn1 cells. (**D** and **E**) Differential expression of genes involved in epithelial mesenchymal transition (EMT) and integrin signaling. (**F**) Heatmap of 51 significantly differentially expressed proteins (after multitesting correction at FDR 5%) determined by reverse phase protein array (RPPA) coincides with transcriptional data. (**G**) GSEA pathway analysis. Pathways are abbreviated as follows: K, KEGG; R, Reactome; H, Hallmark; P, Pathway Interaction Database. (**H**) RNA-Seq data set of TCGA melanomas (*n* = 473). ssGSEA analysis depicting correlation between the upregulated Epng1 gene signature with the proliferative genes (*P* = 1.069 × 10^–6^; OR, 2.61), and the upregulated Epgn3 signature with the invasive genes (*P* = 8.595 × 10^–6^; OR, 2.46).

To investigate the transcriptomes of melanoma cell lines that represent Epgn1 and Epgn3 groups, we first examined the expression of the 122–epigenetic gene signature in 12 BRAF V600 mutant cell lines derived from human primary cutaneous melanomas using a customized NanoString array (Supplemental Table 1.1 and 1.2; supplemental material available online with this article; https://doi.org/10.1172/jci.insight.166611DS1). Unsupervised hierarchical clustering validated 2 groups, Epgn1 (*n* = 6) and Epgn3 (*n* = 6) (Supplemental Figure 1A). We next employed bulk RNA-Seq to examine the transcriptomes of the cell lines identified as Epgn1 (*n* = 3; WM35, YUPEET, WM983A) or Epgn3 (*n* = 2; WM1552C, YUCHIME), as well as primary melanocytes (*n* = 2). Principal component analysis (PCA) revealed the separation of predicted experimental groups (Supplemental Figure 1B). RNA-Seq defined *n* = 4,608 differentially expressed genes between Epgn1 versus Epgn3 subsets (*q* < 0.05, Benjamini-Hochberg procedure and a linear fold-change ± 1.5; Supplemental Table 1.3). Unsupervised hierarchical clustering using epigenetic genes differentially expressed between the groups (*n* = 5) validated the Epgn1 and Epgn3 subsets (Figure 1A). Of importance, we noted that Epgn1/Epgn3 clustering is similar to the gene signature clustering of proliferative and invasive genes underlying phenotype switching described by Hoek et al. (2). Utilizing Hoek’s signature, we were able to cluster these cell lines based on increased expression of proliferative genes (i.e., *SOX10*, *MITF*, *CDK2*) in the Epgn1 and invasive genes (i.e., *EGFR*, *WNT5A*, *PDGFC*, *CDH2*, *ZEB1*) in the Epgn3 groups (Figure 1B). The melanocyte lineage-specific *MITF* — along with other pigmentation pathway genes, many of which also belong to the proliferative gene subset (i.e., *SOX10*, *MLANA*, *PMEL*, *TYR*, *DCT*, *TYRP1*) — were observed as upregulated in the Epgn1 state (Figure 1C). High MITF protein expression in the Epgn1 cells and low-level, if any, expression in the Epgn3 cells was confirmed by Western blotting (Supplemental Figure 1C).

Of interest, we observed increased expression of genes involved in adhesion and remodeling, such as collagen family members (i.e., *COL1A1, COL5A1, COL6A1, COL7A1*) and integrins (i.e., *ITGA1, ITGA2, ITGA3, ITGA5, ITGA6, ITGA8, ITGB10, ITGB1, ITGAV*) in the Epgn3 group (Figure 1, D and E). To validate the transcriptomic data, a reverse-phase protein array (RPPA) analysis was performed. Proteins such as HES1, MDM2, ERBB3, BAP1, PARP1, SOX2, and KIT were upregulated in the Epgn1 cell lines corresponding to the genes observed as upregulated in the Epgn1 cellular state by RNA-Seq. By contrast, proteins such as AXL, STAT3, ITGA2, NRG1, JUN, SMAD1, ANXA7, SERPINE, and EGFR were upregulated in the Epgn3 cell lines corresponding to invasive genes upregulated in this group (Figure 1F and Supplemental Table 1.4). Gene set enrichment analysis (GSEA, KEGG pathways) indicated an upregulation of DNA- and RNA-mediated pathways, including DNA replication, DNA repair, mRNA processing, transcription, mRNA splicing, cell cycle mitosis, and meiosis in the Epgn1 state. Pathways involved in invasion, including epithelial-mesenchymal transition, integrin signaling, angiogenesis, focal adhesion signaling, and inflammatory processes, were upregulated in the Epgn3 group (Figure 1G). To further validate the association between Epng1 and Epng3 signatures with the proliferative and invasive genes underlying phenotype switching, respectively, we examined their correlation using RNA-Seq of The Cancer Genome Atlas (TCGA) of cutaneous melanoma data set (*n* = 473) via single-sample GSEA (ssGSEA). This analysis showed a significant correlation of Epgn1 signature with the proliferative genes (*P* = 1.069 × 10^–6^; OR, 2.61) and Epgn3 signature with the invasive genes (*P* =8.595 × 10^–6^; OR, 2.46; Figure 1H). In summary, we provide evidence that an epigenetic gene signature (Epgn1/Epgn3) is associated with proliferative and invasive signatures (phenotype switching) in primary melanoma cell lines and TCGA melanomas. These data suggest epigenome deregulation underpinning these processes (1).

The GSEA pathway analysis indicated a number of signaling pathways that were not previously characterized in detail as part of the phenotype switching transcriptome. We found genes related to DNA replication, transcription, mRNA processing, DNA repair, cell cycle mitosis, adhesion, remodeling, and integrins, inflammation, adhesion, and hypoxia (Supplemental Figure 2, A–N). In aggregate, these transcriptomic data suggest that signals related to the pigmentation pathway machinery and DNA replication processes dominate in the Epgn1 proliferative cells. In contrast, epithelial-to-mesenchymal transition, integrin, hypoxia, angiogenesis, and proinflammatory signals dominate in the Epgn3 invasive cells.

### An epigenetic gene signature (Epgn1/Epgn3) classifies human primary melanomas into low- versus high-risk groups.

We previously published on the identification of the 122-epigenetic gene classifier (Epgn1/Epgn3) and its association with low- versus high-risk primary melanomas (*n* = 51) (14). Here, we sought to validate the classifier in a larger cohort (*n* = 205) of primary cutaneous melanomas > 1.0 mm in thickness (Supplemental Tables 2.1 and 2.2). We excluded acral and uveal melanomas and those with distant metastasis. The AJCC eighth edition staging system was used for clinical and pathological classification. The new cohort was composed of invasive melanomas with tumor thicknesses greater than 1.0 (>T1; Supplemental Table 2.1), the most difficult group of tumors to prognosticate. The mean, as well as the median tumor thickness, was 3.0 mm. Tumors were in intermediate (T2, 1.01–2.0 mm, *n* = 49; T3, 2.01–4.0 mm, *n* = 91) and thick (T4, >4.0 mm, *n* = 65) thickness categories. The majority were stage II cases (*n* = 110). Overall survival was 67 months (mean) and 57.5 months (median) (alive, *n* = 109; dead, *n* = 80; and unknown, *n* = 16).

**Figure 2:**
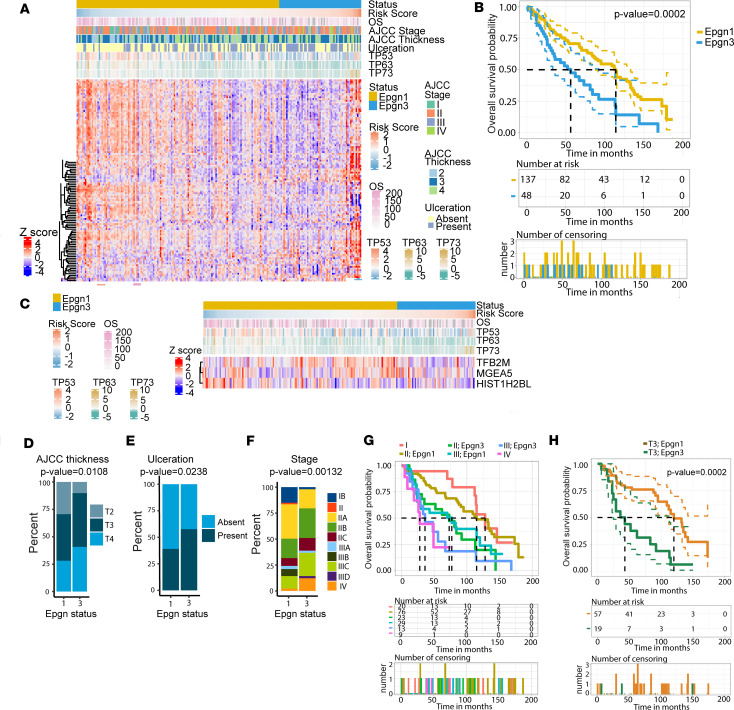
Epgn1/Epgn3 gene signature classifies primary melanoma tumor samples into low- versus high-risk groups. (**A**) Heatmap of a cohort of primary cutaneous melanoma samples (*n* = 205). The heatmap depicts expression of 118 epigenetic genes from our signature along with *TP53* family and housekeeping genes. Each row of the heatmap indicates a differentially expressed gene (*n* = 118), and each column represents a tumor sample (*n* = 205). The status bar indicates the classifier: Epgn1 (gold) and Epgn3 (light blue). The overall survival (OS) risk score, AJCC thickness, ulceration, stage, and OS (number of months) is color-coded as indicated. (**B**) Kaplan-Meier survival curves for Epgn1 (gold) and Epgn3 (blue) subgroups. (**C**) Heatmap showing the minimum number of epigenetic genes (*HIST1H2BL, MGEA5, TFB2M*) that correlated with OS. Correlation with our OS risk score allows for the identification of our 2 groups Epgn1 (gold) and Epgn3 (blue). *TP53, TP63,* and *TP73* gene expression are depicted. Cox regression model was used. (**D**) The AJCC tumor thickness in the Epgn1 group versus the Epgn3 group (*P* = 0.0108). (**E**) Ulceration in the Epgn1 group versus the Epgn3 group (*P* = 0.0238). (**F**) Stage in the Epgn1 group versus the Epgn3 group (*P =* 0.00132). One-way ANOVA test was used (**D**–**F**). (**G**) Kaplan-Meier curve showing the Epgn1/Epgn3 risk classifier in discriminating better versus poor OS by Stage. (**H**) Kaplan-Meier curve showing the Epgn1/Epgn3 risk classifier in discriminating better versus poor OS for patients with T3 tumors (*P* = 0.0002).

RNA was isolated from formalin-fixed paraffin-embedded (FFPE)tumors (*n* = 205). The expression of 118 of 122 epigenetic genes from our original signature (14), along with the *TP53* family and housekeeping genes (Supplemental Table 1.2), was assayed on a custom array using the NanoString platform (Figure 2A). For data analysis, we first cross-interrogated the NanoString platform versus RNA-Seq in classifying the Epgn1/Epgn3 groups using 24 samples from the original study (14) (Supplemental Table 2.3). We validated 78 epigenetic genes of 118 that showed differential expression between the groups at FDR 5% with both technologies (Supplemental Figure 3A and Supplemental Tables 2.4 and 2.5). To classify the new cohort of 205 samples on the NanoString platform, we next developed a melanoma risk score and a classifier that distinguishes tumors as Epgn1 (low-risk) or Epgn3 (high-risk). To identify the epigenetic genes correlating with overall survival (OS) and progression-free survival (PFS), we performed univariate Cox analysis for OS and PFS for each of the 118 epigenetics genes in the 205 samples. We identified 21 nominally significant genes associated with OS and 12 nominally significant genes associated with PFS (Supplemental Tables 2.6 and 2.7). Since there are partial correlations between gene expression, a 50–cross-validated relaxed LASSO regression analysis as well as a stepwise procedure was conducted to reduce the number of genes and select those with a nonzero coefficient in a multivariate Cox regression model. The multivariate Cox OS model, the 50–cross-validated relaxed LASSO analysis, as well as the stepwise procedure, identified a combination of 3 genes — *HIST1H2BL, MGEA5,* and *TFB2M* — as our melanoma risk score (Supplemental Table 2.8).

In order to transition our melanoma risk score into a classifier, we identified the optimal threshold at 0.18 that gives us the lowest confusion matrix from the original discovery cohort data set. Using this optimal cutoff threshold (0.18), our melanoma OS risk classifier in the new 205 samples showed an OS of 56 months for patients in the Epgn3 group and 114 months for patients in the Epgn1 group (*P* = 2 × 10^–4^; Figure 2B) and predicted a PFS of 29 months for the Epgn3 group and 75 months for the Epgn1 group (*P* = 1 × 10^–4^; Supplemental Figure 3B). Additionally, the OS risk score and the Epgn1/Epgn3 classifier correlated with the *TP53* family of genes (*TP53, TP63,* and *TP73*), similar to our previous report (Figure 2C and Supplemental Table 2.9) (14). When AJCC parameters were examined, Epgn3 tumors were thicker (Figure 2D), more likely to be ulcerated (Figure 2E), and had higher AJCC staging (Figure 2F). While tumor thickness, age at diagnosis, and survival status were statistically significant between the 2 groups, sex and primary tumor location were not (Supplemental Figure 3, C–G). Next, we added AJCC clinical staging (stages I–III) or tumor thickness categories (T2–T4) to our OS risk score to determine if collinearity exists between the AJCC parameters and our OS risk model. We found that our OS risk classifier composed of 3 genes is complementary to the AJCC parameters (Supplemental Table 2.10). Indeed, we improved the discrimination between better OS and poor OS mainly for patients in stage II and stage III disease (*P* = 0.0002 and *P* = 0.001; Figure 2G) and with T3 tumor thickness category (*P* = 0.0002; Figure 2H). We also noted that patients classified as Epgn1/stage III have similar OS as those in the Epgn3/stage II group (Figure 2G).

Overall, these results validate our Epgn1/Epgn3 classifier (122 or 3 genes) in distinguishing low- versus high-risk cases in a new independent cohort of primary cutaneous melanomas consisting of tumor thickness (>1.0 mm) and clinical stage categories (majority in stages II and III) that are the most challenging to prognosticate. The reduced number of genes (3 genes) and the OS risk score may be employed as biomarkers for Epgn1/Epgn3 and may be relevant to phenotype switching in early disease.

### Deregulation of H3K27 acetylation is a dominating epigenetic mechanism in Epgn1 low-risk and Epgn3 high-risk states (or during melanoma cell dedifferentiation).

**Figure 3:**
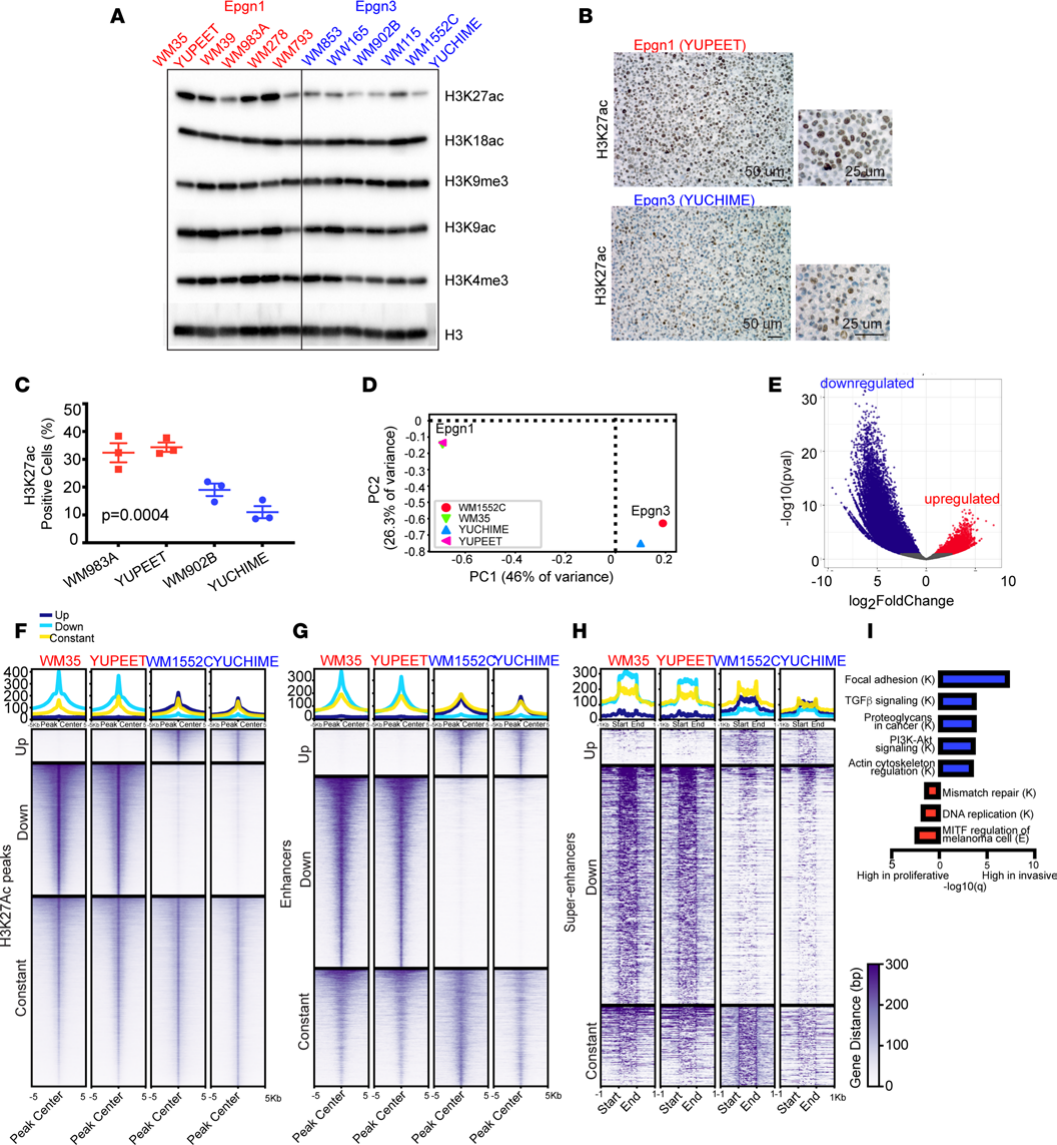
Reorganization of super-enhancer and enhancer landscape in Epgn1/Epgn3 melanoma cells. (**A**) Immunoblotting of the chromatin fraction of Epgn1 and Epgn3 cells for H3K27ac, H3K18ac, H3K9me3, H3K9ac, and H3K4me3. Total H3 indicates loading. (**B**) IHC of H3K27ac in YUPEET, a representative Epgn1 cell line and YUCHIME, a representative Epgn3 cell line. Scale bars: 50 μm (left), 25 μm (right). (**C**) Quantification of data generated in 2 Epgn1 cell lines WM983A and YUPEET (red) as compared with 2 Epgn3 cell lines WM902B and YUCHIME (light blue). *P* = 0.0004. One-way ANOVA test was used. (**D** and **E**) ChIP-Seq for H3K27ac using representative Epgn1 cells, WM35 and YUPEET, and representative Epgn3 cells, WM1552C and YUCHIME. PCA plot indicating the separation of groups. Volcano plot showing a total of 85,858 peaks from differential peak analysis: 7,924 upregulated peaks in the Epgn3 group, and 31,511 downregulated peaks in the Epgn3 group (upregulated in the Epgn1 group). (**F**) Heatmap of differential peak analysis. Data are presented on ± 5 kb around the peak center. DiffBind package was used. (**G**) Heatmap of differential enhancer analysis. Data are centered on ± 5 kb window. A total of 2,220 significant enhancers were identified in the Epgn3 cell lines and 9,074 significant enhancers were identified in the Epgn1 cell lines. (**H**) Heatmap of differential super-enhancer analysis. Data are shown ± 1 kb upstream and downstream of the super-enhancer. A total of 83 and 533 significant super-enhancers were identified in the Epgn3 and Epgn1 cell lines, respectively. ROSE algorithm was used. (**I**) GSEA (KEGG pathway) analysis identifies super-enhancers and enhancers associated with functional pathways. K, KEGG; E, Elsevier.

Since Epgn1/Epgn3 groups modeled in the cell lines (Figure 1) and tissues (Figure 1H and Figure 2) are classified based on this 122-epigenetic gene signature that suggests epigenome deregulation, we sought to understand *cis*-regulatory elements of gene regulation underlying phenotype switching. We first examined the major histone marks associated with either active or repressed transcriptional states in Epgn1 and Epgn3 cells by Western blotting of the chromatin fraction. We noted a consistent decreased histone 3 lysine 27 acetylation (H3K27ac) in the Epgn3 cells (Figure 3A). Other histone marks that were assayed (H3K9ac, H3K18ac, H3K4me3, and H3K9me3 in Figure 3A; H3K4me1, H3K9me2, H3K27me3, and H3K36me3 in Supplemental Figure 4) did not show major consistent differences between the 2 cell line groups. IHC confirmed low levels of H3K27ac in the Epgn3 cell lines compared with the Epgn1 lines (Figure 3, B and C).

We next sought to investigate the super-enhancer and enhancer landscape in the Epgn1/Epgn3 cell lines using H3K27 acetylation — a marker of active enhancers. We employed ChIP-Seq for H3K27ac using Epgn1 (WM35 and YUPEET) and Epgn3 cell lines (WM1552C and YUCHIME). PCA revealed the separation of experimental groups based on their chromatin activity profile, similar to the clustering observed by RNA-Seq (Figure 3D). Differential peak analysis defined a total of 85,858 peaks: 7,924 (9%) upregulated peaks in the Epgn3 group and 31,511 (37%) downregulated peaks in the Epgn3 group (upregulated in the Epgn1 group) (Figure 3E). A total of 2,220 Epgn3-specific enhancers and 83 super-enhancers were identified in the Epgn3 cell lines, and 9,074 Epgn1-specific enhancers and 533 super-enhancers were identified in the Epgn1 cell lines (Figure 3, F–H) (Supplemental Tables 3.1–3.4.), suggesting a reorganization of the enhancer landscape in Epgn3 versus Epgn1 states. Next, we associated super-enhancers and enhancers with nearby genes; we identified 308 and 2,792 Epgn3 genes, respectively, and 1,418 and 3,179 Epgn1 genes, respectively (Supplemental Tables 3.1–3.4). GSEA (KEGG pathway) analysis showed that nearby genes associated with super-enhancers/enhancers were involved in cell motility and invasion-related pathways such as focal adhesion, PI3K/Akt signaling, and actin cytoskeleton in the Epgn3/invasive cell lines. By contrast, those associated with MITF regulation and DNA-mediated processes were observed in the Epgn1/proliferative cell lines (Figure 3I). Taken together, these findings suggest that substantial reorganization of the epigenetic landscape underlie Epgn1 low-risk/proliferative and Epgn3 high-risk/invasive cellular states.

### Super-enhancers are associated with melanocytic lineage-specific genes in the Epgn1 low-risk/proliferative melanoma cells lines and invasion genes in the Epgn3 high-risk/invasive cell lines.

**Figure 4:**
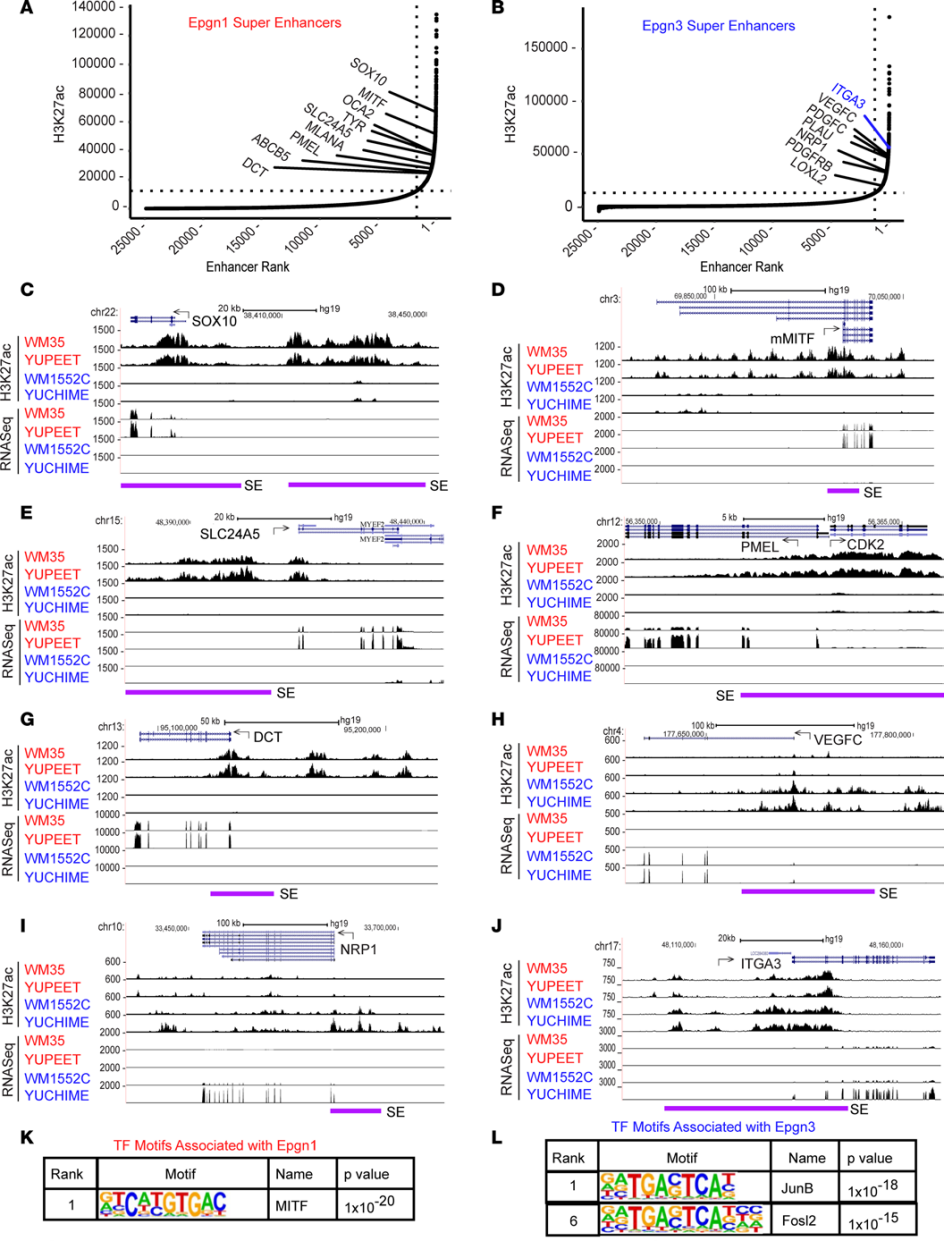
Super-enhancers are associated with MITF lineage genes in Epgn1/proliferative cell lines and cell motility genes in Epgn3/invasive cell lines. (**A**) Ranked order of H3K27ac normalized reads at super-enhancer and enhancer loci in Epgn1 cell lines. Super-enhancers that associated with genes belonging to the MITF lineage specific pigmentation pathway in the Epgn1 cell lines are shown. (**B**) Ranked order of H3K27ac normalized reads at super-enhancer and enhancer loci in Epgn3 cell lines. Super-enhancers associated with invasive genes in the Epgn3 cell lines are shown. ROSE algorithm was used. (**C**–**G**) UCSC genome browser captures of H3K27ac and RNA-Seq enrichment profiles are shown for selected Epgn1 genes and their super-enhancers including *SOX10* (**C**); the melanocyte specific MITF isoform, *mMITF* (**D**); *SLC24A5* (**E**); *PMEL* and *CDK2* (**F**); and *DCT* (**G**). (**H**–**J**) H3K27ac traces along with corresponding RNA-Seq peaks are shown for selected Epgn3 genes including *VEGFC* (**H**), *NRP1* (**I**), and *ITGA3* (**J**). Super-enhancer regions are denoted by purple line. (**K**) The top motif and corresponding transcription factors associated with Epgn1 super-enhancers. (**L**) The top motifs and corresponding transcription factors associated with Epgn3 super-enhancers. Fisher’s exact test was used (**K** and **L**).

Super-enhancers have the potential to activate oncogenic transcription and tend to be associated with genes that control and define cell identity. We identified several super-enhancers associated with genes belonging to the MITF-dependent pigmentation pathway in the Epgn1 cell lines (Figure 4A). In contrast, super-enhancers associated with invasive genes were identified in the Epgn3 cell lines (Figure 4B). We show representative examples of super-enhancer elements mapping upstream of the Epgn1 genes, such as *SOX10* (Figure 4C), *MITF* (Figure 4D), *SLC24A5* (Figure 4E), *PMEL* (Figure 4F), and *DCT* (Figure 4G), which have higher H3K27ac super-enhancer peaks corresponding to higher RNA-Seq gene expression in the Epgn1 cell lines than in the Epgn3 cell lines. In contrast, we show examples of super-enhancers that map upstream of the Epgn3 genes, including *VEGFC* (Figure 4H), *NRP1* (Figure 4I), and *ITGA3* (Figure 4J), which have higher H3K27ac super-enhancer peaks corresponding to higher RNA-Seq gene expression in the Epgn3 cell lines than in the Epgn1 cell lines.

Alterations in TF activity regulate gene expression programs. We next performed TF motif analysis in the Epgn1/Epgn3-specific enhancers (using HOMER v.4.11 suites) that identified MITF as the top-ranked motif associated with Epgn1 enhancers (Figure 4K and Supplemental Table 3.5) and AP1 motifs associated with Epgn3 enhancers (Figure 4L and Supplemental Table 3.6). Our findings are consistent with previous reports identifying MITF and AP-1/TEADs as regulators of the proliferative and invasive cellular states in melanoma (7, 15), while recognizing some novel TF genes that have not previously been linked to these processes. In aggregate, through ChIP-Seq of H3K27ac and RNA-Seq analyses, we show that differential regulation of super-enhancers/enhancers and corresponding changes in the activity of the master regulatory genes drive dynamic transcriptional changes in distinct cellular states; Epgn1 low-risk/proliferative cellular state or Epgn3 high-risk/invasive state.

### ITGA3 is regulated by a super-enhancer region in Epgn3 cell lines and acts as a suppressor of invasiveness.

**Figure 5:**
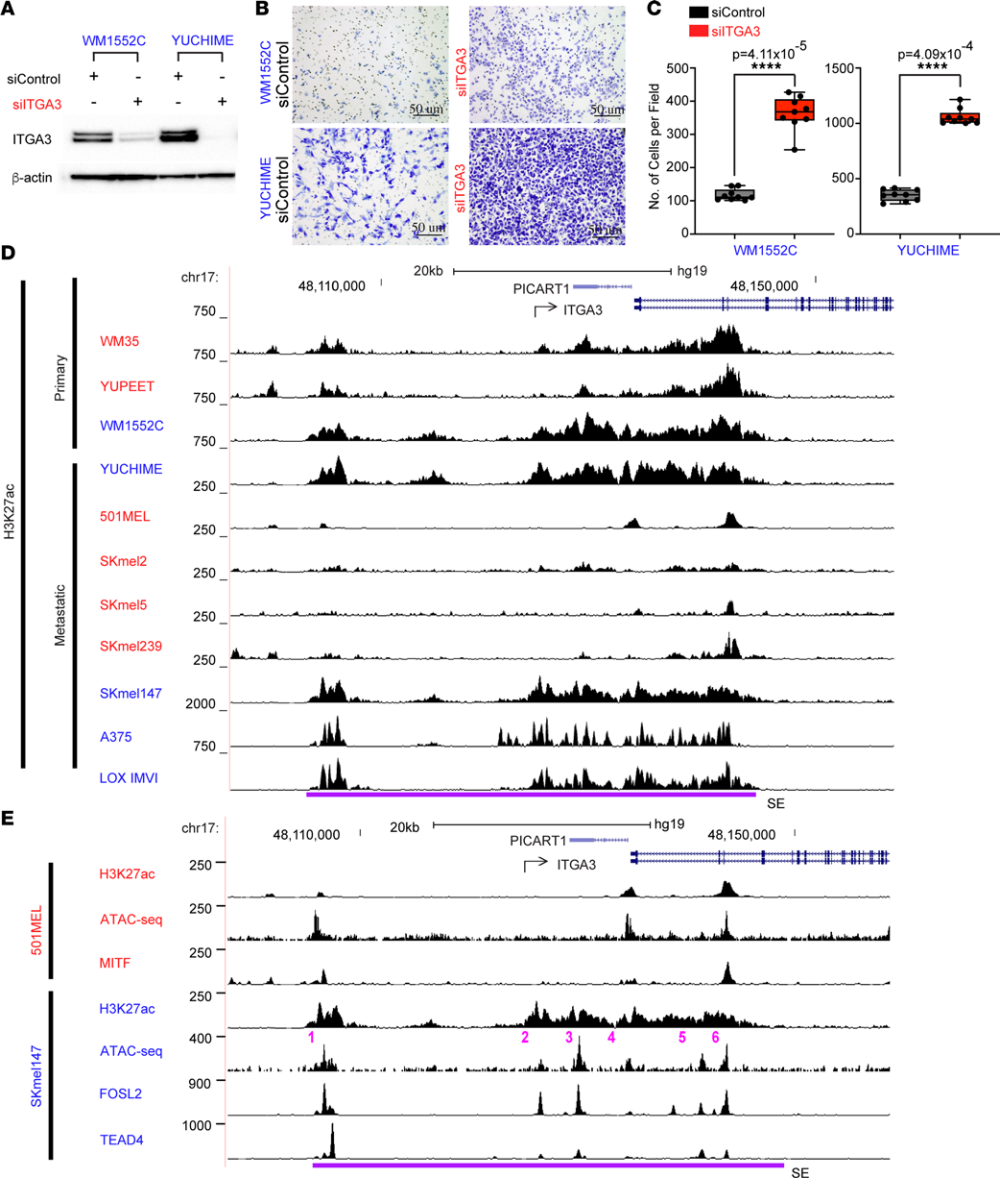
ITGA3 suppresses invasion and is associated with super-enhancer–mediated regulation in Epgn3 cells. (**A**) ITGA3 immunoblot of lysates following transfection with si*Control* or si*ITGA3*. Actin was used as a loading control. (**B**) Representative examples of cells following invasion in a matrigel coated Boyden chamber model. Scale bars: 50 μm. (**C**) Quantification of invasion assay shown in **B**. Two groups are compared: si*Control* and si*ITGA3* in the Epgn3 primary melanoma cell lines. *P* = 4.11 × 10^–5^ for WM1552C and *P* = 4.09 × 10^–4^ for YUCHIME. Mann Whitney *U* test was used. (**D**) H3K27ac ChIP-Seq peaks in the Epgn1 primary and metastatic cell lines (red) and in the Epgn3 primary and metastatic cell lines (blue). Super-enhancer region is denoted with a purple line. (**E**) H3K27ac, ATAC-Seq, and MITF ChIP-Seq peaks in the Epgn1 cell line 501MEL. H3K27ac, ATAC-Seq, FOSL2, and TEAD4 ChIP-Seq peaks in the Epgn3 cell line SKmel147.

To assess the functional effect of Epgn3 genes associated with super-enhancers, we selected 5 Epgn3 gene candidates not previously implicated in melanoma pathogenesis and tested them in a loss-of-function in vitro invasion assay. We transiently transfected primary melanoma cell lines, WM1552C and YUCHIME, with separate siRNA pools against each of the 5 candidates and performed in vitro Boyden chamber invasion assay. The loss of one of the candidates, *ITGA3*, displayed a robust phenotype and showed a significantly increased invasion of cells into the Boyden chambers (Figure 5, A–C). Based on this effect of *ITGA3* on cellular invasion and its super-enhancer profile in the Epgn3 invasive cell lines, we further investigated its role in melanoma biology.

*ITGA3* is one of the genes for phenotype switching described by Hoek et al*.* (2) (Figure 1B), and in this study, we identified it as an integrin transcriptionally upregulated in the Epgn3 invasive cell lines (Figure 1E). We found a large super-enhancer region extending from the gene body to about 20 Kb upstream in the MITF-low Epgn3 cell lines (WM1552C and YUCHIME), whereas MITF-high Epgn1 cell lines (WM35 and YUPEET) show a markedly reduced H3K27ac levels correlating with lower ITGA3 expression levels (Figure 5D). The neighboring gene, *PICART1*, is not expressed in our cell lines and, thus, is unlikely to be regulated by this super-enhancer. *ITGA3* has higher H3K27ac super-enhancer peaks corresponding to higher RNA-Seq gene expression peaks in the Epgn3 cell lines than the Epgn1 cell lines. Additionally, using published H3K27ac ChIP-Seq data, we identified this super-enhancer in the MITF-low metastatic melanoma cell lines (SKmel147, A375, and LOX IMVI) and found loss of H3K27ac in the MITF-high metastatic melanoma cell lines (501MEL, SKmel2, SKmel5, and SKmel239) (7, 8, 15) (Figure 5D).

Based on ChIP-Seq and ATAC-Seq analyses, we identified constituent enhancers within the ITGA3 super-enhancer (Figure 5E). We further performed motif analysis within a 200 bp window around the ATAC peak summit and identified the TF AP1 and its family member TEAD1 among the top 5 ranked TFs corresponding to the constituent enhancers (Supplemental Figure 5) (15). Enrichment profiles for H3K27ac, ATAC-Seq, FOSL2 ChIP-Seq, and TEAD4 ChIP-Seq in the SKmel147 cell line further support the involvement of the AP1 and TEAD TFs in regulating the ITGA3 super-enhancer (Figure 5E). Regions of overlap between the ATAC-Seq, FOSL2, and TEAD4 peaks within the H3K27ac super-enhancer region indicated that the *ITGA3* super-enhancer is bound by FOSL2 and TEAD4, thus suggesting that AP1 may be regulating *ITGA3* (Figure 5E) and coinciding with our finding of AP1 transcription family members as the top-ranked motifs associated with Epgn3 enhancers (Figure 4L). In contrast, in the MITF-high cell line 501MEL, the absence of *MITF* ChIP-Seq peaks indicated that the *ITGA3* super-enhancer is not bound by *MITF* (Figure 5E).

**Figure 6:**
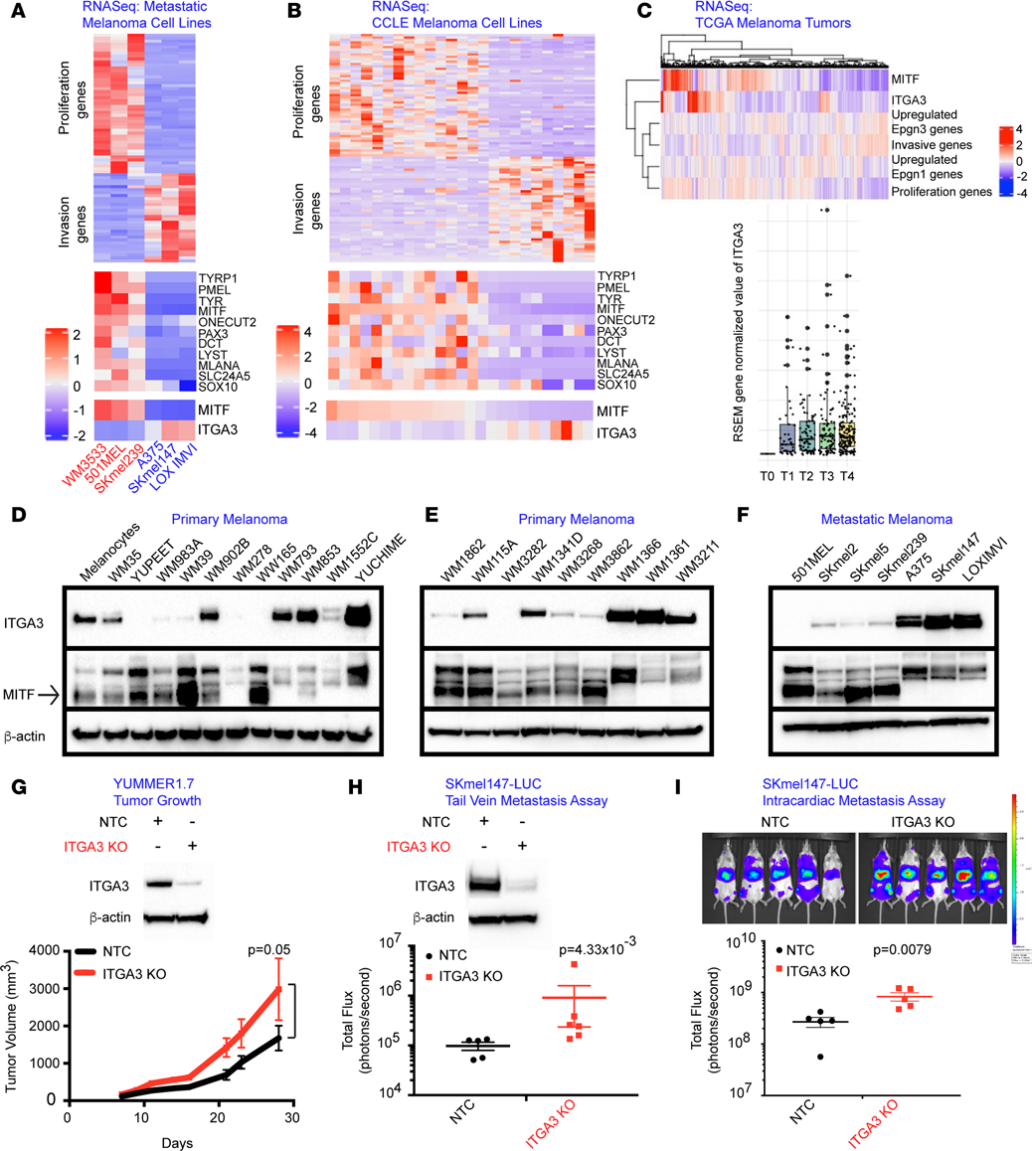
ITGA3 is overexpressed in Epgn3 high-risk melanomas and negatively regulates an invasive phenotype. (**A**) Heatmaps of proliferation, invasion, and pigment pathway genes *MITF* and *ITGA3* in the metastatic melanoma cell lines. (**B**) Heatmaps of proliferation, invasion, and pigment pathway genes *MITF* and *ITGA3* in the CCLE melanoma cell lines. (**C**) ssGSEA depicting correlations between proliferation, invasion, Epgn1, and Epgn3 gene signatures with *MITF* and *ITGA3* (upper panel). Dot plot of the *ITGA3* expression for AJCC tumor stages, T0-T4 (TCGA melanoma data set, lower panel). (**D**–**F**) Western blotting for ITGA3, MITF, and actin in the primary (**D** and **E**) and the metastatic melanoma cell lines (**F**). (**G**) Immunoblotting of ITGA3 and actin in the nontargeting control (NTC) and ITGA3-KO YUMMER1.7 cells. Tumor growth assay following the injection of the NTC and ITGA3-KO YUMMER1.7 cells in mice. *P* = 0.05. (**H** and **I**) Immunoblotting of ITGA3 and actin in the NTC and ITGA3 KO SKmel147 luciferase cells (**H**). Tail vein (**H**) and intracardiac metastasis (**I**) assays following the injection of the nontargeting control NTC and ITGA3 KO cells in mice, P = 4.33 × 10^–3^ and P = 0.0079, respectively. Mann Whitney *U* test was used (**G**–**I**).

At the transcriptional level, metastatic cell lines designated as Epgn1 (*n* = 3) based on high expression of *MITF* and other proliferation and pigmentation pathway genes showed low expression of *ITGA3,* whereas metastatic cell lines identified as Epgn3 (*n* = 3), based on low *MITF* expression and high expression of invasion genes, showed high *ITGA3* transcript levels. There was anticorrelation between *MITF* and *ITGA3* (*P* = 0.0125, Figure 6A and Supplemental Figure 6A). Similar findings were noted in Cancer Cell Line Encyclopedia (CCLE) melanoma cell lines (*n* = 25) and an anticorrelation between *MITF* and *ITGA3* was present (*P* = 0.002, OR, –0.54, Figure 6B). We next examined bulk RNA-Seq data from TCGA melanoma tumor samples (*n* = 473), and the relationship between the proliferative, invasive, Epgn1, and Epgn3 signatures with *MITF* and *ITGA3* (Figure 6C). We identified a correlation between proliferation signature and *MITF* (0.69; *P* < 2.2 × 10^–16^) and the Epgn1 signature (0.1; *P* = 0.02), and we identified an anticorrelation between the proliferation and Epgn3 signatures (–0.385; *P* < 2.2 × 10^–16^). We found an anticorrelation between the invasion signature and *MITF* (–0.61; *P* < 2.2 × 10^–16^) and the Epgn1 signature (–0.41; *P* < 2.2 × 10^–16^) and a correlation between the invasion and Epgn3 signatures (0.17; *P* = 2.9 × 10^–4^) and *ITGA3* (0.145, *P* = 1.6 × 10^–3^). Of clinical importance, we identified increased *ITGA3* gene expression in AJCC T4 versus T1 tumors (*P* = 0.06) (Figure 6C). To further substantiate the transcriptomic data in cell lines at the protein level, Western blotting for ITGA3 in a panel of primary and metastatic melanoma cells was performed, which confirmed the anticorrelation of ITGA3 with MITF (Figure 6, D–F, and Supplemental Figure 6, B–D). Thus, both at transcript and protein levels, MITF-high cells showed low levels of ITGA3, whereas MITF-low cells expressed high ITGA3 levels (Figure 6, A–F, and Supplemental Table 1.1).

We next examined the role of *ITGA3* on tumorigenesis and tumor cell invasiveness in vivo. Utilizing CRISPR-Cas9 genome editing, we generated models of *ITGA3* loss in the murine melanoma YUMMER1.7 (Braf^V600E/WT^, Pten^–/–^, Cdkn2^–/–^) cell line and in the human metastatic melanoma SKmel147 cell line. Immunoblotting was performed to assess ITGA3 protein levels. YUMMER1.7 melanoma cells stably expressing a nontargeted control (NTC) or *ITGA3* KO were injected s.c. into the flanks of syngeneic C57BL/6J mice, and tumor volume was measured. Mice injected with *ITGA3*-KO YUMMER1.7 cells showed a significant increase in tumor growth compared with mice injected with the NTC (Figure 6G), indicating that loss of ITGA3 confers increased tumorigenesis. We next examined a metastatic human melanoma cell line SKmel147 in experimental metastasis models. SKmel147 luciferase–expressing *ITGA3* NTC versus KO cells were delivered via tail vein or intracardiac injection of immunocompromised mice (NOD/SCID/IL2γR^−/−^) and analyzed by IVIS imaging, which showed increased seeding and metastatic burden to the lungs or the liver, respectively, in *ITGA3-*KO cells (Figure 6, H and I). These data provide evidence that ITGA3 negatively regulates tumor cell invasiveness in the in vivo setting. In aggregate, these findings suggest that ITGA3 is associated with super-enhancer–mediated regulation during melanoma cell dedifferentiation (phenotype switching) and in Epgn1/Epgn3 states. Its expression anticorrelates with MITF, and its upregulation and overexpression in the Epgn3 high-risk/invasive cellular state are suppressive for invasiveness, tumor growth, and metastatic potential.

## Discussion

Super-enhancers are clusters of enhancers that are binding sites for master transcription regulators enriched for active histone modifications (H3K27ac, H3K4me1) and the Mediator complex (16). Super-enhancer–driven genes are expressed at higher levels than those under the control of regular enhancers and are associated with developmentally regulated genes that specify cell identity (16). Here, we found MITF, a melanocytic cell lineage TF, as well as its regulators and targets associated with H3K27ac-marked super-enhancers and high gene expression in the Epgn1 low-risk/proliferative cellular state that is reorganized in the Epgn3 high-risk invasive state. While MITF and other TFs are essential for the development and homeostasis of the melanocyte lineage, they also play important roles during melanoma initiation and progression, and they are well characterized during phenotype switching. Our findings corroborate with epigenome regulation of lineage-specific genes and highlight this mechanism of gene regulation during melanoma cell dedifferentiation or phenotype switching as dominating — a mechanism that has not been described previously in this context. Additionally, we identified many super-enhancers and enhancers regulating the transcriptional output of the cell differentially modulated in distinct cellular states and potentially during the reversal of the switch from one cellular state to another, demonstrating the essential role of epigenome reprogramming. Mechanisms of differential enhancer landscape of proliferative/invasive states need further studies; transcriptional factors (MITF or others) binding to the super-enhancers, histone acetylation (acetyl transferases and deacetyl transferases), and the role of the BET proteins (BRD4) that may be regulating the process could be further dissected. In particular, MITF as the master regulator of the melanocyte lineage and the extent of its involvement in super-enhancer regulation in this process could be examined. Dissecting the epigenome in-depth in the future remains of interest, as epigenetic drugs to target melanoma cells in different cellular states may enhance therapeutic targeting efforts.

Melanomas fall into 4 genomic categories based on driver mutations: BRAF (50%), NRAS (25%), or NF1 (15%) mutant and triple WT (17). Mutational status predicts responses to BRAF and MEK inhibitors rather than correlating strongly with biological behavior. Transcriptomic subtyping, however, has been promising in predicting patient outcomes. Our Epgn1/Epgn3 classifier separates primary cutaneous melanomas into low- versus high-risk categories, correlates with AJCC staging parameters, and is an independent predictor for OS. We found that the Epgn1/Epgn3 classifier correlates with dedifferentiation or phenotype switching (proliferative or invasive cellular states) based on RNA-Seq of primary melanoma cell lines and TCGA melanomas. Therapeutic targeting of phenotype switching has been challenging due to plasticity, the reversible switch from one state to another, and resistance due to the selection of subpopulations of cells upon treatment. We were able to reduce our Epgn1/Epgn3 classifier from 122 genes to 3 genes; this classifier may be helpful as a biomarker for Epgn1/Epgn3 states, dedifferentiation, or phenotype switching for therapy efforts in the future.

Integrins, cellular adhesion receptors for the extracellular matrix, have been implicated in essentially every step of cancer progression from primary tumor formation to metastasis (18). The role of integrins in cancer cell progression and metastasis is dependent on the tumor type and disease state. Here, we examined *ITGA3,* which encodes the α3 subunit that dimerizes with β1 to form the laminin-binding integrin α3β1. Roles for α3β1 in both promoting and suppressing tumorigenesis and metastasis have been described in several cancer types; however, its role in melanoma is largely unknown (19). There are opposing data on ITGA3 expression during tumor progression in melanoma. One study reported reduced *ITGA3* transcripts in melanoma cell lines and tumors of regional and distant metastasis compared with primary disease (20). Other studies showed an opposite relationship: a correlation of ITGA3 protein expression with tumor thickness (21), higher ITGA3 expression on metastatic melanoma cell lines, and higher migration rates as compared with primary melanoma cell lines (22). The role of ITGA3 in the context of phenotype switching in melanoma has never been described, to our knowledge. Our study provides evidence that *ITGA3* is a gene involved during dedifferentiation or phenotype switching via super-enhancer regulation. ITGA3 is upregulated and overexpressed in the Epgn3 invasive cellular state and shows an inverse relationship with MITF. Our in vitro and in vivo studies show that ITGA3 is a negative regulator of invasiveness, tumor progression, and metastatic potential in this context — roles that have not yet been described in melanoma. While this protective mechanism and how ITGA3 acts as a braker for invasiveness require further studies, the paper highlights integrins playing essential roles during the dedifferentiation process in primary cutaneous melanoma.

## Methods

### Sex as a biological variable.

Our study examined NSG female mice with experiments using the SKmel147 human cell line. It is expected that the findings are relevant for male mice (Figure 6, H and I). Our study examined C57BL/6J male mice with experiments using the YUMMER 1.7 cells (Figure 6G), since the parental cell line was generated in male mice. It is expected that the findings are relevant in female mice.

### Patient samples and cell lines.

A cohort of patients with primary melanomas of the skin (*n* = 205) with fully annotated clinical and pathological parameters was collected from 4 academic institutions (Supplemental Table 2; Icahn School of Medicine at Mount Sinai, Moffitt Cancer Center, Columbia University Medical Center, and University Hospital of Zurich). Primary tumors stored as FFPE were retrieved and compiled, and histology was reviewed. Primary melanoma cell lines were obtained from the Wistar Institute (M. Herlyn, Philadelphia, Pennsylvania, USA): WM35, WM39, WM115A, WM278, WM793, WM853, WM902B, WM983A, WM1552C, WM1341D, WM1361, WM1366, WM1862, WM3211, WM3268, WM3282, and WM3862. The following BRAF^V600^ mutant melanoma cell lines derived from primary tumors were obtained from Yale University (R. Halaban, New Haven, Connecticut, USA): YUPEET (BRAF^V600E^), YUCHIME (BRAF^V600K^), and WW165 (BRAF^V600K^). Human melanocytes were purchased from 2 sources: (a) Invitrogen primary melanocytes, HEMn-LP (Human Epidermal Melanocytes neonatal, lightly pigmented donor) (Thermo Fisher Scientific, C0025C), and (b) human epidermal melanocytes from White neonatal foreskin (Cell Applications, 104-05n). Metastatic cell lines SKmel2, SKmel5, and A375 were obtained from ATCC. Metastatic cell line LOX IMVI was obtained from Sigma-Aldrich. SKmel147 and SKmel239 were obtained from Memorial Sloan Kettering Cancer Center (New York, New York, USA). Metastatic cell line 501MEL was obtained from Yale University. Murine melanoma cells YUMMER1.7 were obtained from Yale University (M. Bosenberg). HEK293T cells used for virus production were obtained from ATCC.

### RNA-Seq of melanoma cell lines and data analysis.

Total RNA from cells cultured in triplicate was extracted using the RNeasy Mini Kit (Qiagen). The RIN of each sample was determined using the 2100 Bioanalyzer instrument (Agilent Technologies) and quantity was determined using Qubit (Thermo Fisher Scientific). Total RNA was subjected to ribosomal RNA depletion using the Ribo-Zero Gold kit (Illumina). The resulting RNA samples were used as input for library construction using the Illumina TruSeq Total RNA library preparation kit (Illumina). The libraries were then sequenced on the NextSeq500 system (Illumina) using the 75 bp paired-end protocol. The sequencing was performed at the Oncological Sciences Core Facility of the Icahn School of Medicine at Mount Sinai. Raw sequencing reads were aligned to the human genome (GRCh38.74) using STAR (23) (19.25 × 10^6^ uniquely mapped reads per sample) and aligned reads assigned to transcript features using HTSeq (24). Read alignment quality measures and feature assessment were examined by FastQC (http://www.bioinformatics.babraham.ac.uk/projects/fastqc/) and RSeQC (25). Raw feature counts were normalized, and differential expression analysis was performed using DESeq2 and used Benjamini-Hochberg multitesting correction at 5 % to identify significant genes (26). Differential expression rank order was utilized for subsequent GSEA (27), performed using the fgsea package (https://bioconductor.org/packages/release/bioc/html/fgsea.html) in R. Gene sets queried included the Hallmark and Canonical pathway collections available through the Molecular Signatures Database (MSigDB) (28).

### RPPA.

Cellular proteins were denatured by 1% SDS (with β-mercaptoethanol) and diluted in five 2-fold serial dilutions in the dilution lysis buffer. Serially diluted lysates were arrayed on nitrocellulose-coated slides (Grace Bio-Labs) by Aushon 2470 Arrayer (Aushon BioSystems) at MD Anderson Cancer Center. Total 5,808 array spots were arranged on each slide, including the spots corresponding to serially diluted (a) standard lysates and (b) positive and negative controls prepared from mixed cell lysates or dilution buffer, respectively. Each slide was probed with a validated primary antibody plus a biotin-conjugated secondary antibody. The signal obtained was amplified using a Dako Cytomation–Catalyzed system (Dako) and visualized by DAB colorimetric reaction. The slides were scanned, analyzed, and quantified using customized software to generate spot intensity. Each dilution curve was fitted with a logistic model (http://bioinformatics.mdanderson.org/OOMPA). This fits a single curve using all the samples (i.e., dilution series) on a slide with the signal intensity as the response variable, and the dilution steps are independent variables. The fitted curve is plotted with the signal intensities — both observed and fitted — on the *y* axis and the log_2_ concentration of proteins on the *x* axis for diagnostic purposes. The protein concentrations of each set of slides were then normalized for protein loading. A correction factor was calculated by (a) median centering across samples of all antibody experiments and (b) median centering across antibodies for each sample.

### ssGSEA for TCGA SKCM skin cutaneous melanomas.

ssGSEA is a gene set variation analysis (GSVA) that provides an estimate of pathway activity by calculating a gene set enrichment score per sample (29). We analyzed RNA-Seq data of TCGA skin cutaneous melanomas (SKCM) skin cutaneous melanoma using the R package called GSVA (version 1.44.2) (30) with the Hoek signature of invasion and proliferation and the signature of Epgn1 (genes identified to be positively differentially expressed in our previous study accessible in Supplemental Table 1.2) and Epgn3 (genes identified to be negatively differentially expressed in our previous study accessible in Supplemental Table 1.2) (2, 14). We assigned for each sample the status of invasion, proliferation, Epgn1, and Epgn3 when we identified an enrichment of their signatures through ssGSEA. We observed that some samples could have both positive or negative invasion and proliferation signatures as well as both positive or negative Epgn1 and Epgn3 signatures, showing that they are not mutually exclusive signatures. We then performed an enrichment analysis to identify enrichment of invasion and Epgn3 as well as proliferation and Epgn1 in the TCGA SKCM samples.

### Gene expression analysis on tissue samples.

Total RNA was extracted from four 10 μm curls of FFPE tissue with the RecoverAll Total Nucleic Acid Isolation Kit (Invitrogen). The RNA integrity number (RIN) of each sample was determined using the 2100 Bioanalyzer instrument (Agilent Technologies), and quantity was determined using Qubit (Thermo Fisher Scientific). Gene expression analysis on patient tissue samples as well as melanoma cell lines was performed utilizing the NanoString nCounter platform (NanoString Technologies). We assayed 200 ng of total RNA utilizing a custom code set of genes consisting of 118 of our 122 epigenetic gene signature that we reported previously (14), TP53 family genes, and housekeeping genes (Supplemental Table 1.2). The nSolver analysis software version 4.0 (NanoString Technologies) was used to extract raw digital counts of expression, check the quality of the data, and generate heatmaps.

### Prognostic risk score model to identify low-risk (Epgn1) versus high-risk (Epgn3) groups.

In an effort to determine a minimal gene set that was able to discriminate between our 2 cohorts (Epgn1 versus Epgn3), we began by identifying the genes correlated with OS and PFS by performing the univariate Cox analysis on the 118 genes with *P* < 0.05 and using normalized data by NanoString’s method. To select genes with a strong prognostic value, eliminate the correlation between genes, create a prognostic risk score model, and prevent overfitting of the final model, we then evaluated gene expression signatures by 2 reduction methods, relaxed least absolute shrinkage and section operator (LASSO) with 50-fold cross-validation using “glmnet” R library (version 4.0-2) and stepwise variable selection procedure (with iterations between the “forward” and “backward” steps) using “My.stepwise” R library (version 0.1.0). The coefficient of each gene estimated in the final model was used as a coefficient for the prognostic risk score model, such as

Risk Score =


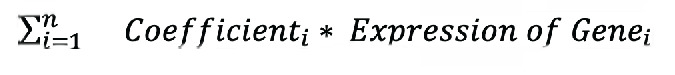


Based on the prognostic risk score model, we computed the risk score for each patient in 205 new samples and 24 JCI samples (Supplemental Table 2.3 and ref. 14). To test the accuracy of the models and identify the threshold to split the cohort into low-risk (Epgn1-like) and high-risk (Epgn3-line) groups, we used the gene expression of our 24 individuals previously annotated in our original discovery cohort. Kaplan-Meier survival curves were used to evaluate whether there was a significant difference between the low-risk and high-risk groups by a log-rank test with *P* < 0.05. A confusion matrix was established to describe the performance of a classification model on JCI data (Supplemental Table 2.3 and ref. 14), which we previously annotated as Epgn1, Epgn2, or Epgn3.

### Whole cell protein extraction and immunoblotting.

Whole cell protein lysates were made from cell pellets, resuspended in 1× cell lysis buffer (Cell Signaling Technology) supplemented with protease inhibitors (Roche) and phosphate inhibitors (Roche), incubated at 4°C for 30 minutes with shaking, and centrifuged for 10 minutes at 24,375*g* at 4°C. Protein concentrations were determined by Qubit, and lysates were adjusted with lysis buffer to normalize the protein concentrations. Proteins (25–50 μg/lane) were subjected to SDS-PAGE, transferred to a PVDF membrane by standard Western blotting conditions, blocked in 5% milk/PBST for 1 hour, and incubated with primary antibodies (1:1,000 in blocking buffer) overnight at 4°C. The secondary antibody (1:2,500 in blocking buffer) was incubated at room temperature for 1 hour before standard, enhanced chemiluminescence detection. Blots were stripped with Restore Plus Western blot stripping buffer (Thermo Fisher Scientific), reblocked, and reprobed using an appropriate loading control.

### Chromatin extraction for immunoblotting of histone modifications.

Cells were resuspended in 1 mL Buffer A (10 mM HEPES [pH 7.9] [Thermo Fisher Scientific], 10 mM KCl [Invitrogen], 1.5 mM MgCl_2_ [Invitrogen], 0.34M sucrose [Thermo Fisher Scientific], and 10% glycerol [Thermo Fisher Scientific] with 1 mM DTT [Invitrogen], protease inhibitors [Roche], and 0.1% Triton X-100 [Thermo Fisher Scientific]). Following a 10-minute incubation on ice, cells were spun at 4,000 rpm at 4°C for 5 minutes. The supernatant (cytoplasm) was removed, and the nuclear pellet was washed with 1 mL buffer A and 1 mM DTT. Cells were spun at 4,000 rpm at 4 °C for 5 minutes, and nuclei were resuspended in a salt-free buffer containing 3 mM EDTA and 0.2 mM EGTA for 45 minutes with shaking. Following a final spin at 1,500*g* at 4°C for 5 minutes, the resulting chromatin pellet was solubilized in Laemelli buffer with 50 mM DTT and boiled prior to loading on a gel for immunoblotting.

### IHC.

Slides were baked overnight at 37°C, followed by deparaffinization in xylene and rehydration in ethanol. Heat-induced epitope retrieval was performed at pH 9 (Dako Target Retrieval Solution, S2367) for 30 minutes, pigmentation was removed using Pretreatment Solutions A and B (Polysciences), and endogenous peroxidase was removed with 3% H_2_O_2_. Serum-Free Protein Block (Dako, X909) was added for 30 minutes, followed by 30-minute incubation with the primary antibody H3K27ac (Abcam, 177178) diluted in Antibody Diluent with Background Reducing Components (Dako, S3022). Slides were incubated with the anti-rabbit secondary antibody for 30 minutes and streptavidin/HRP (Dako, P0397) for 30 minutes. Slides were developed using SignalStain DAB Chromagen. Images were taken on a Nikon Eclipse Ci microscope and analyzed using NIS-Elements BR Analysis software.

### ChIP-Seq.

ChIP samples were processed as previously described (15), with several modifications. For H3K27ac ChIP (Abcam, 177178), 10 million cells per sample were cross-linked with 1% formaldehyde for 10 minutes at room temperature. Cross-linked cells were quenched with 0.125M glycine for 5 minutes at room temperature, followed by pelleting at 400*g* for 3 minutes at 4°C. Cells were washed once with ice-cold PBS and resuspended at 10 million cells in 500 μL of cell lysis buffer (10 mM Tris [pH 8], 10 mM NaCl, 0.2% NP-40, 100 nM PMSF, supplemented with protease inhibitors), followed by 15 minutes of incubation on ice. Next, cells were centrifuged at 400*g* for 5 minutes at 4°C and resuspended in 500 μL cold nuclear lysis buffer (50 mM Tris [pH 8], 10 mM EDTA, 1% SDS, 100 nM PMSF, supplemented with protease inhibitors) and incubated on ice for 10 minutes. Cells were sonicated for 22 cycles — 30 seconds on, 30 seconds off — at low intensity in a Bioruptor sonicator (Diagenode). After sonication, samples were centrifuged at 13,000*g* for 10 minutes at 4°C, and the supernatant-containing chromatin was diluted 1:4 with i.p. Dilution Buffer (20 mM Tris [pH 8], 2 mM EDTA, 150 mM NaCl, 1% Triton-X, 0.01% SDS, 100 nM PMSF, supplemented with protease inhibitors). Chromatin was precleared with protein A+G (MilliporeSigma) magnetic beads preconjugated with rabbit IgG for 2 hours at 4°C. After preclearing, 50 μL of chromatin was saved as input control. *Drosophila* spike-in chromatin was added to the precleared chromatin in equal amounts across samples to allow sample to sample comparison. A preconjugated antibody (5 μg, H3K27ac, Abcam, 177178) was added to the precleared chromatin and rotated overnight at 4°C. Following overnight incubation, beads were washed once with cold i.p. Wash I Buffer (20 mM Tris [pH 8], 2 mM EDTA, 50 mM NaCl, 1% Triton-X, 0.1% SDS, 100 nM PMSF, supplemented with protease inhibitors), twice with cold High-Salt Buffer (20 mM Tris [pH 8], 2 mM EDTA, 500 mM NaCl, 1% Triton-X, 0.01% SDS, 100 nM PMSF, supplemented with protease inhibitors), once with cold i.p. Wash II Buffer (10 mM Tris [pH 8], 1 mM EDTA, 0.25 LiCl, 1% NP-40, 1% deoxycholic acid, 100 nM PMSF supplemented with protease inhibitors), and twice with cold TE buffer (5 mM Tris [pH 7.4], 1 mM EDTA). DNA was eluted twice in 100 μL elution buffer (1% SDS, 100 mM NaHCO3) at 65°C for 30 minutes at 800 rpm in a thermomixer. For input, 130 μL TE buffer, 12 μL 5M NaCl, 20 μL 10% SDS, and 2 μL of RNase A (10 mg/mL) were added, followed by overnight incubation at 65°C to reverse cross-link. For the ChIP samples, 12 μL of 5M NaCl and 2 μL of RNase A (10 mg/mL) was added, followed by overnight incubation at 65˚C to reverse cross-link. Proteinase K (4 μL at 20 mg/mL) was added, and samples were incubated for 2 hours at 42°C. DNA was purified using the Qiagen MinElute PCR Purification Kit following the manufacturer’s protocol. DNA sequencing libraries were prepared using the NEBNext Ultra II DNA Library Prep Kit for Illumina (NEB). Libraries were analyzed for concentration by Qubit and samples were run on an Agilent2000 DNA HS Bioanalyzer Chip. The libraries were then sequenced on the NextSeq500 system (Illumina) using a 75-bp single-end protocol. The sequencing was performed at the Oncological Sciences Core Facility of the Icahn School of Medicine at Mount Sinai.

### ChIP-Seq alignment and peak calling.

Reads were aligned to the human reference genome hg19 using Bowtie v1.1.2 (31) with parameters –l 40 –n 2 –best –k 1 –m 1, and read quality was assessed using fastQC (32). Duplicate reads were removed with PICARD v2.2.4 (Broad Institute). Binary alignment maps (BAM) files were generated with samtools v1.9 (33) and used in downstream analysis. MACS2 v2.1.0 (34) was used to call significant peaks (H3K27ac, *q* < 1 × 10^–11^). Peaks within ENCODE blacklisted regions were removed. Coverage tracks were generated from BAM files using deepTools 3.2.1 (35) bamCoverage with parameters –scaleFactor X and –binsize 10. The normalization scale factor was calculated for each sample based on the *Drosophila* deduplicated uniquely aligned reads as previously described (36).

### ChIP-Seq differential enrichment analysis.

Significant H3K27ac peaks for all 4 cell lines (WM35, WM1552C, YUCHIME, YUPEET) were merged to generate a master regions file. The DiffBind package (37) was used to identify differentially bound sites within the master regions file. BAM files of ChIP input and *Drosophila* H2AV ChIP were included for normalization. Significant differentially enriched regions were determined using *P* < 0.05. Differential enhancers and super-enhancers were called using the same methodology described above, utilizing a master enhancer or super-enhancer regions file.

### Enhancer and super-enhancer calling and gene associations, and TF motif analysis.

Enhancers and super-enhancers were called based on H3K27ac enrichment using the ROSE algorithm (Rank Ordering of Super-enhancers) (16, 38) with a parameter stitching distance of 12.5 kb. ChIP inputs were used as a control. Differential enhancers/super-enhancers were associated with positively correlated promoters of differentially expressed genes within a genomic range of ± 1,000 kb. TF binding motif enrichments of differential enhancers were generated using the HOMER v.4.11 suites (39). De novo motifs were identified within a 200 bp window around the peak center with the following parameters findMotifsGenome.pl hg19 -size 200 with the default HOMER-generated background regions. TF binding predictions of the ITGA3 super-enhancer were performed on the intersections of the super-enhancer region and ATAC-Seq summits from melanoma cell line SKmel147 (15). TF binding predictions were identified within a 200 bp window around an ATAC peak summit utilizing the Transcription Factor Affinity Prediction Web Tools (40) with the parameter’s matrix jaspar_vertebrates and background model:human_promoters.

### RNA-Seq of cell lines transfected with siRNA.

siRNA constructs were obtained from Horizon (ON-TARGETplus Human siRNA SMARTPool). Transient transfections were performed in 6-well tissue culture plates using 5 μL siRNA (20 μM) and 5 μL DharmaFECT1 transfection reagent per well. Transfection media were replaced after 7 hours, and cell lysates were harvested 72 hours following transfection. We first assessed the quality of paired-end reads with FASTQC (v0.11.8). Next, we filtered reads with BBDUK from BBTOOLS (v37.53) to remove adapters, and known artifacts, and quality trimmed (Phred quality score < 10). Reads that became too short after trimming (*n* < 60 bp) were discarded. Singleton reads (i.e., reads whose mate has been discarded) were not retained. We estimated the transcript-level quantification of cleaned RNA-Seq data using SALMON (v1.0.0) by a quasimapping on *Homo sapiens* transcriptome GRCh38_gencode.v22. We evaluated the gene-level quantification using the tximport library and converted 22,231 human gene expressions. Differential expression was assessed for cell cultures transfected with siRNA constructs (si*Control* versus si*ITGA3*) using the R DESeq2 library (v1.81.1) (26). We called differential expression between 2 conditions when the adjusted *P* value was at 5% and log_2_ fold change was more than 1.

### Proliferation and invasion assays.

Cells were seeded in 6-well tissue culture plates (160,000 cells per well) in quadruplicate for harvesting at each time point. At each harvest time point (24, 48, 72, and 96 hours after cell seeding), 4 wells per cell line were trypsinized and counted using the Countess automated cell counter (Invitrogen). Cells were transfected with pooled siRNA against *ITGA3* for 48 hours. On the day of cell seeding, invasion plates with Matrigel-coated inserts (Corning, 354480) were equilibrated by adding cell culture medium into the wells and inserts and were incubated for 2 hours at 37°C. Cells were collected, counted, and resuspended in a serum-free medium, and 500,000 cells were seeded into each insert in a final volume of 500 μL. A complete growth medium with 20% FBS was inserted as a chemoattractant in the bottom well. Seeded cells were incubated for 24 hours at 37°C. For analysis, the medium in the inserts was aspirated and the upper layer of the membrane was cleaned with cotton swabs to remove the cells that failed to migrate and the matrigel. Following fixation with MeOH and staining with crystal violet, inserts were removed and mounted on a glass slide. Image acquisition of the membranes with the invaded cells was made on a Nikon Eclipse Ci microscope, and cell counts were determined manually using ImageJ (NIH).

### Association of ITGA3 gene expression and clinical data from TCGA SKCM.

We analyzed the bulk RNA-Seq and clinical data of TCGA SKCM from the Broad Institute GDAC Firehose (https://gdac.broadinstitute.org/) and performed Mann-Whitney *U* test between different tumor stages.

### Generation of luciferase-expressing CRISPR KO cell lines.

Stable luciferase expression was established in SKmel147 cells by transfection with the MSCV luciferase PGK-hygro plasmid (Addgene, plasmid 18782) using the Transduce IT lentivirus transduction reagent (Mirus, 6620). Following a 2-week selection with hygromycin B (Invitrogen), the cells were expanded, and luciferase expression was confirmed by bioluminescence imaging. To generate ITGA3 KO YUMMER1.7 (Braf^V600E/WT^, Pten^–/–^ Cdkn2^–/–^) and SKmel147 cell lines, the *ITGA3* sgRNAs were cloned into the Cas9 containing lentiCRISPRv2 (Addgene, 52961) vector system following the published protocol (41, 42). To produce lentiviral particles, HEK293T cells at 80% confluency in a 10 cm tissue culture dish were cotransfected with 5 μg of lentiviral expression constructs, 3.75 μg of psPAX2 (Addgene, 12260), and 1.25 μg pMD2.G (Addgene, 12259) vectors using the TransIT-Lenti Reagent (Mirus, 6650) transfection reagent. Cell-free supernatant was harvested at 48 hours after transfection and was used to transduce YUMMER1.7 and SKmel147 cells (Mirus, 6620). Cells were selected with puromycin (2 μg/mL) following lentiviral infection. KO of ITGA3 was validated by Western blot. We used the clone for in vivo experiments, resulting in completely abolished ITGA3 expression.

### Animal experiments.

Experimental protocols were approved by the IACUC of the Icahn School of Medicine at Mount Sinai and New York University. Six-week-old male C57BL/6J mice and 6-week-old female NOD/SCID/IL2γR^−/−^ mice (The Jackson Laboratory, catalog 05557) were used for in vivo studies. To study tumor growth, 1 × 10^6^ YUMMER1.7 murine melanoma cells were injected s.c. into the flanks of each C57BL/6J mouse. Tumor volumes were calculated using the following equation: 0.5 × l × w^2^. To examine lung metastasis, 1.5 × 10^5^ SKmel147 human melanoma cells were i.v. injected into the lateral tail vein of each NOD/SCID/IL2γR^−/−^ mouse. For the analysis of liver metastasis, 5 × 10^4^ SKmel147 human melanoma cells were delivered into each NOD/SCID/IL2γR^−/−^ mouse by intracardiac injection. Bioluminescence imaging was performed using the Xenogen IVIS 200 (Perkin-Elmer) once per week until the experimental end point. Images were quantified using Living Image software. The mice were anesthetized with 2.5% isoflurane prior to imaging and then injected with 150 mg/kg D-luciferin i.p. (Perkin-Elmer). Exposure time was adjusted to avoid pixel saturation. A total bioluminescence flux (photons/second) was calculated for each region of interest (ROI).

### Data availability.

All omics data are under Super Series at GEO (GSE198432). The RNA-Seq files of the experiments are available at GEO (GSE198425 for Next-Generation Sequencing of Primary Melanocytes, Epgn1, and Epgn3 Melanoma Cell lines; GSE198426 for siRNA ITGA3/control in SKmel147; and GSE198427 for siRNA ITGA3/control in YUCHIME). Nanostring data for human samples and cell lines are available at GEO (GSE198429, GSE198430, and GSE198431). ChIP-Seq data are available at GEO (GSE197235). The RPPA data of our 15 melanoma cell lines are available at GEO (GSE198428). Values for all data points in graphs are reported in the Supporting Data Values file.

### Statistics.

RNA-Seq experiments were performed with 3 replicates. A 2-tailed unpaired *t* test was used when comparing 2 groups, 1- or 2-way ANOVA was used for multiple comparisons, and either Mann Whitney *U* test or log-rank test was used for survival analysis. Statistical analyses were performed using R version 4.0.2 (2020-06-22). Differentially expressed genes identified by Deseq2 are significant if *q* < 0.05 (Benjamini-Hochberg multitesting correction). We used Fisher’s exact test to analyze contingency tables.

### Study approval.

Deidentified melanoma tissue samples were processed as approved by our IRB protocols. Animal experiments were performed after obtaining IACUC approval from Icahn School of Medicine at Mount Sinai and NYU Grossman School of Medicine.

## Author contributions

KM, TCM, and JTC conceived the project. KM performed the laboratory experiments and TCM performed the bioinformatics and statistical experiments. CL, RD, YS, JLM, VKS, and GD contributed tissue samples and clinically annotated data of the patient cohorts. REP provided advice for the bioinformatics design and experimentation. CBN performed the ChIP-Seq data analysis. DH and EB provided oversight for the ChIP-Seq experiments and data analysis. MH and JX assisted with the in vivo experiments. KM, TCM, CBN, and JTC wrote the manuscript. JTC supervised the entire study. All authors read and accepted the manuscript.

## Supplementary Material

Supplemental data

Unedited blot and gel images

Supplemental table 1

Supplemental table 2

Supplemental table 3

Supplemental table 4

Supporting data values

## Version 1 02/06/2024

In-Press Preview

## Version 2 03/22/2024

Electronic publication
