# Supplementary material for: Differential histone acetylation and super-enhancer regulation underlie melanoma cell dedifferentiation
Source: JCI Insight. 2024 Feb 6;9(6):e166611. doi: 10.1172/jci.insight.166611 (PMC11063936; doi:10.1172/jci.insight.166611)
Supplement: Supplemental data [file jciinsight-9-166611-s218.pdf]

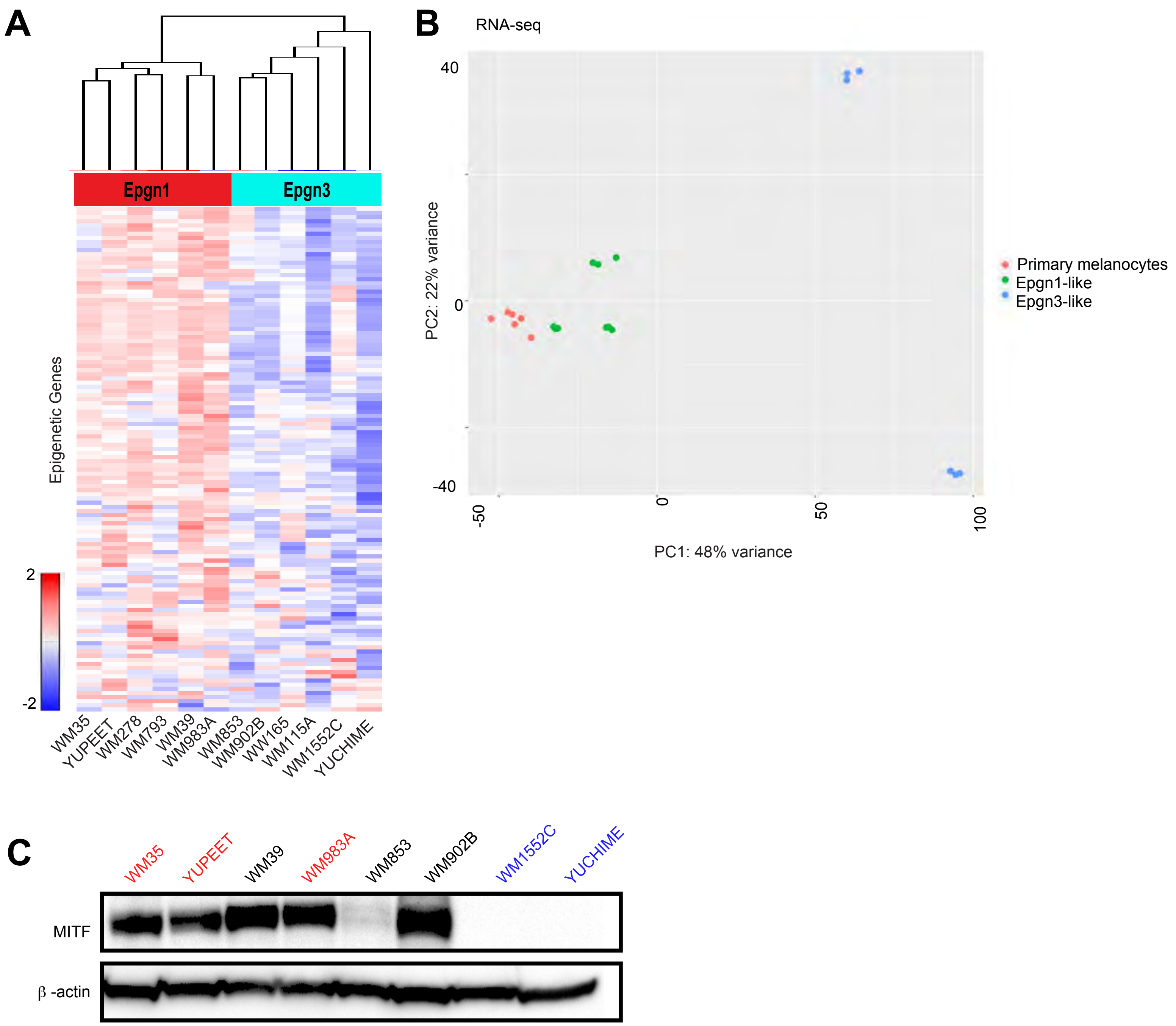

Supplemental Figure 1. Epigenetic gene signature (Epgn1/Epgn3) classifies primary melanoma cell lines into two groups. (A) Unsupervised hierarchical clustering of 122-epigenetic genes using a custom NanoString array identifies Epgn1 and Epgn3 groups in 12 BRAFV600 mutant primary melanoma cell lines. Each row of the heatmap indicates a differentially expressed gene and each column represents a BRAFV600 mutant cell line (n=12). The heatmap is color-coded on the basis of z-scores. The top bar indicates the cellular subtype; Epgn1 (red) and Epgn3 (blue). **(B)** Principal component analysis (PCA) of bulk RNA sequencing examined the transcriptomes of representative cell lines identified as Epgn1 (n=3) or Epgn3 (n=2) and primary melanocytes (n=2). **(C)** Western blot of MITF expression in BRAFV600 mutant cell lines. Cell lines identified by RNA sequencing as Epgn1 are highlighted in red (WM35, YUPEET, and WM983A) and cell lines identified as Epgn3 (WM1552C and YUCHIME) are highlighted in blue.

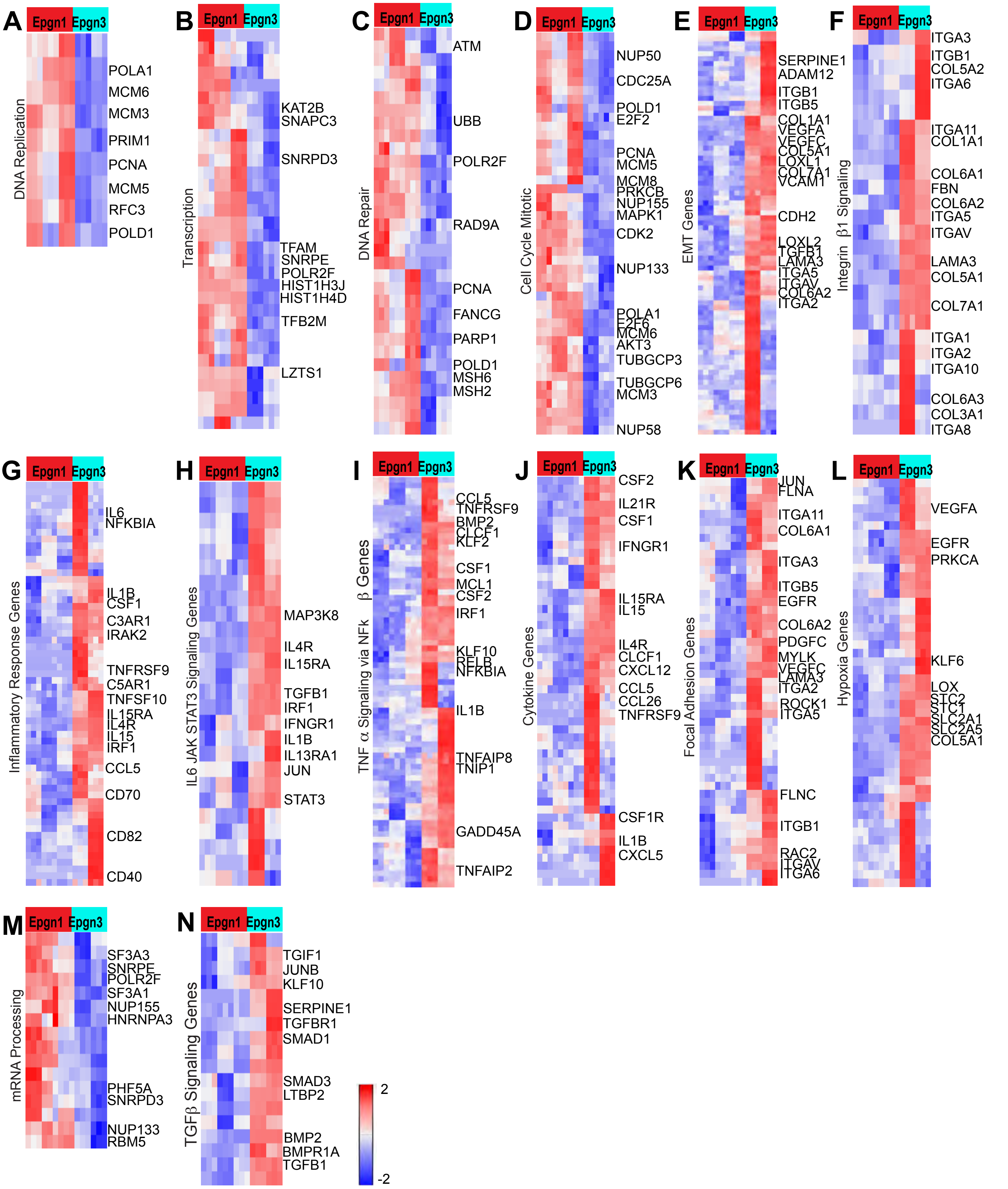

Supplemental Figure 2. Differentially expressed genes in primary melanoma cell lines classified as Epng1 or Epgn3. Bulk RNA sequencing of primary melanoma cell lines designated as Epgn1 (WM35, YUPEET, WM983A) or Epgn3 (WM1552C, YUCHIME) identified n=4,608 differentially expressed genes. GSEA (KEGG pathway) analysis found several deregulated pathways **(A-N).**

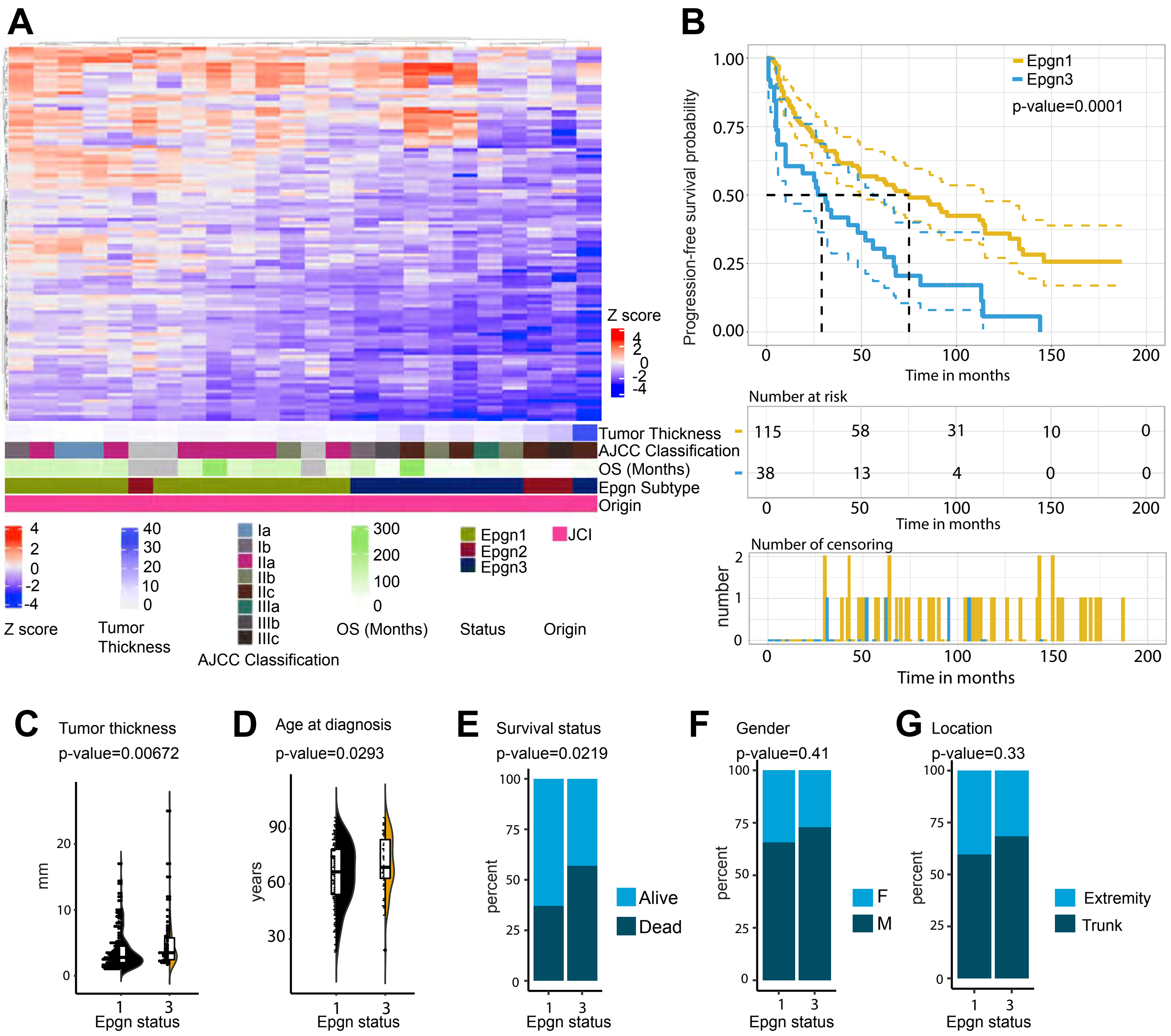

Supplemental Figure 3. Epigenetic gene signature (Epgn1/Epgn3) classifies human primary melanomas into low- versus high-risk groups using NanoString platform. (A) Unsupervised hierarchical clustering of 24 samples from our original cohort [Badal et. al.] using 78 genes (out of 122 genes) measured by a customized NanoString array identified two main clusters corresponding to Epgn1 and Epgn3 groups. Each row of the heat-map indicates a differentially expressed gene (n=78) and each column represents a tumor sample (n=24). Tumor thickness, AJCC stage, OS (number of months), and epigenetic status are color-coded as indicated. **(B)** Our melanoma overall risk survival classifier on the 205 samples shows progression-free survival for patients from the Epgn3 group versus progression-free survival for patients from the Epgn1 group (p-value=1x10^{4}). **(C-G)** Tumor thickness, age at diagnosis, survival status, gender and location as compared between Epgn1 and Epgn3 groups. Wilcoxon Rank Sum (C and D) and Fisher exact tests (E-G) were used.

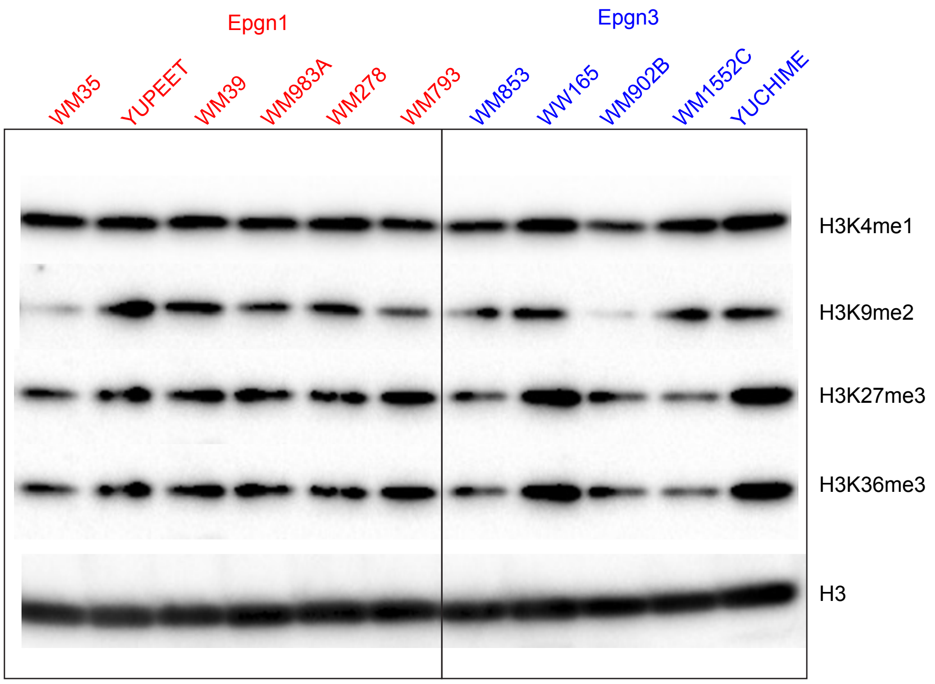

Supplemental Figure 4. Expression of histone marks in primary melanoma cells classified as Epgn1 versus Epgn3. Profiling of Epgn1/3 cell lines for H3K4me1, H3K9me2, H3K27me3, and H3K36me3 by Western blotting of the chromatin fraction. H3 is shown as a loading control.

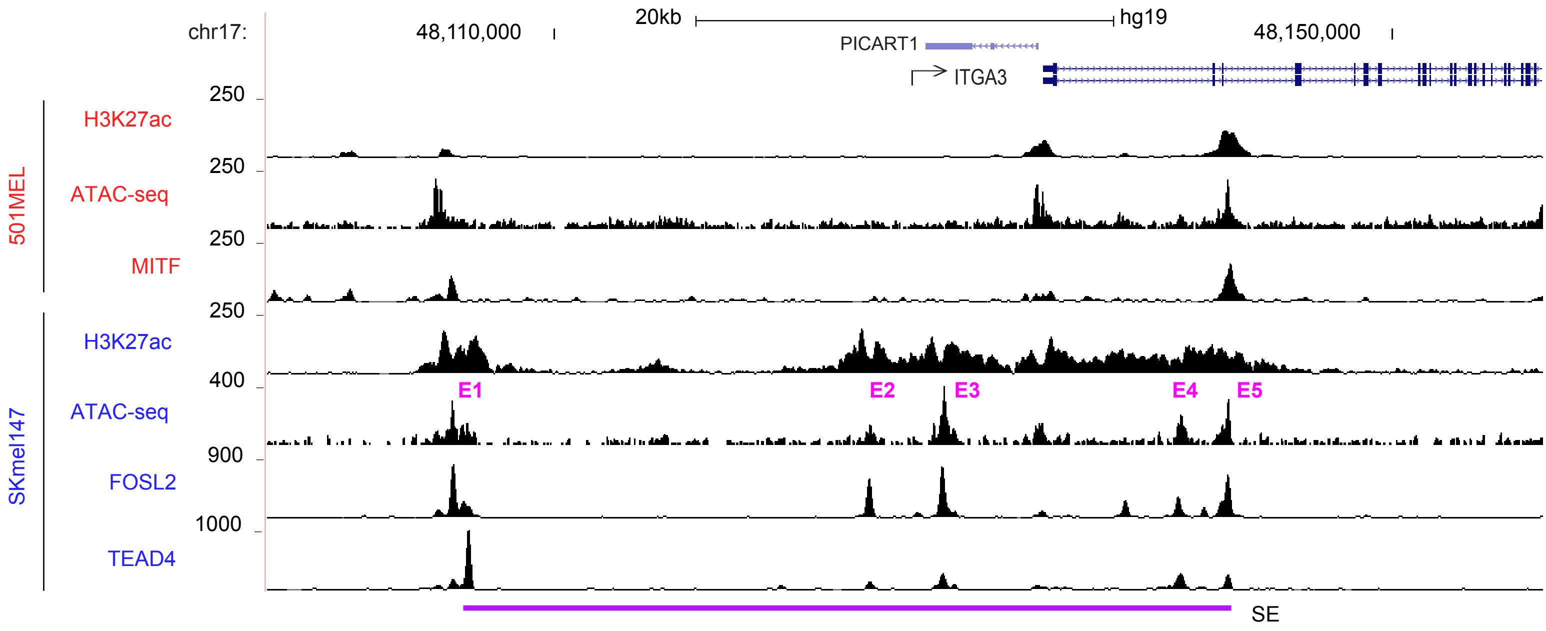

TF associations with enhancers

E1

Rank	Name	p value
1	REST	$8.96x10^{-3}$
2	Myf	$1.74x10^{-2}$
3	RREB1	$2.96x10^{-2}$
4	ELK1	$3.48x10^{-2}$
5	**AP1**	$4.48x10^{-2}$

E2

Rank	Name	p value
1	**AP1**	$1.24x10^{-2}$
2	SPI1	$1.27x10^{-2}$
3	CREB1	$3.8x10^{-2}$
4	SP1	$5.17x10^{-2}$
5	IRF2	$5.27x10^{-2}$

E3

Rank	Name	p value
1	NFκB	$3.06x10^{-3}$
2	**AP1**	$4.13x10^{-3}$
3	NFE2L2	$4.81x10^{-3}$
4	FEV	$1.65x10^{-2}$
5	znf143	$1.71x10^{-2}$

E4

Rank	Name	p value
1	TP53	$2.03x10^{-3}$
2	**TEAD1**	$6.63x10^{-3}$
3	EBF1	$3.3x10^{-2}$
4	Nr2e3	$4.54x10^{-2}$
5	Stat3	$4.66x10^{-2}$

E5

Rank	Name	p value
1	SPI1	$1.41x10^{-2}$
2	**AP1**	$1.45x10^{-2}$
3	ELK1	$3.21x10^{-2}$
4	GABPA	$3.25x10^{-2}$
5	Stat3	$4.58x10^{-2}$

Supplemental Figure 5. Transcription factor binding predictions with the ITGA3 super-enhancer in Epgn3 cell lines. H3K27ac, ATAC-seq and MITF ChIP-seq peaks in the MITF-high cell line 501MEL (red). H3K27ac, ATAC-seq, FOSL2, and TEAD4 ChIP-seq peaks in the MITF-low cell line SKmel147 (blue). Five constituent enhancers (E1-5) identified within the super-enhancer region in the SKmel147 cell line (indicated by the purple line). The top predicted transciption factors (TFs) around the five ATAC peak summits are indicated in the respective tables. AP1 complex family members are highlighted in blue. HOMER v.4.11 suite and Fisher exact test was used.

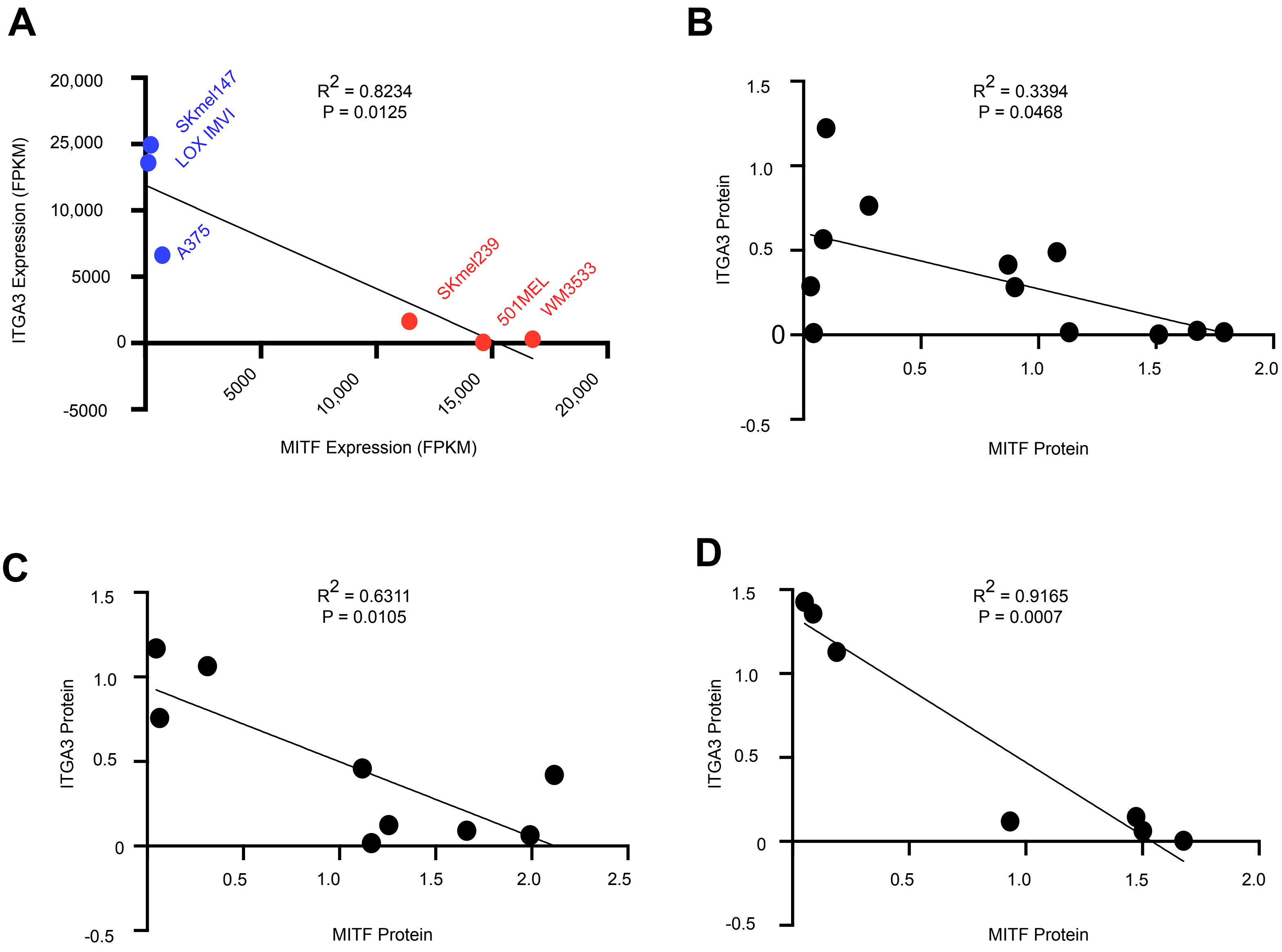

Supplemental Figure 6. Anti-correlation between ITGA3 and MITF expression at transcript and protein level. (A) Linear regression plot showing anti-correlation between ITGA3 and MITF expression at the transcript level. FPKM values from RNA sequencing are plotted. **(B)** Quantification of Western blot for primary melanoma cell lines shown in Figure 6C. Linear regression plot of MITF protein (x-axis) normalized to β–actin versus ITGA3 protein (y-axis) normalized to β–actin. **(C)** Quantification of Western blot for primary melanoma cell lines shown in Figure 6D. Linear regression plot of MITF protein (x-axis) normalized to β–actin versus ITGA3 protein (y-axis) normalized to β–actin. **(D)** Quantification of Western blot for metastatic melanoma cell lines shown in Figure 6E. Linear regression plot of MITF protein (x-axis) normalized to β–actin versus ITGA3 protein (y-axis) normalized to β–actin.
