# Supplementary material for: Differential histone acetylation and super-enhancer regulation underlie melanoma cell dedifferentiation
Source: JCI Insight. 2024 Feb 6;9(6):e166611. doi: 10.1172/jci.insight.166611 (PMC11063936; doi:10.1172/jci.insight.166611)
Supplement: Unedited blot and gel images [file jciinsight-9-166611-s219.pdf]

Figure 3A
H3K27ac

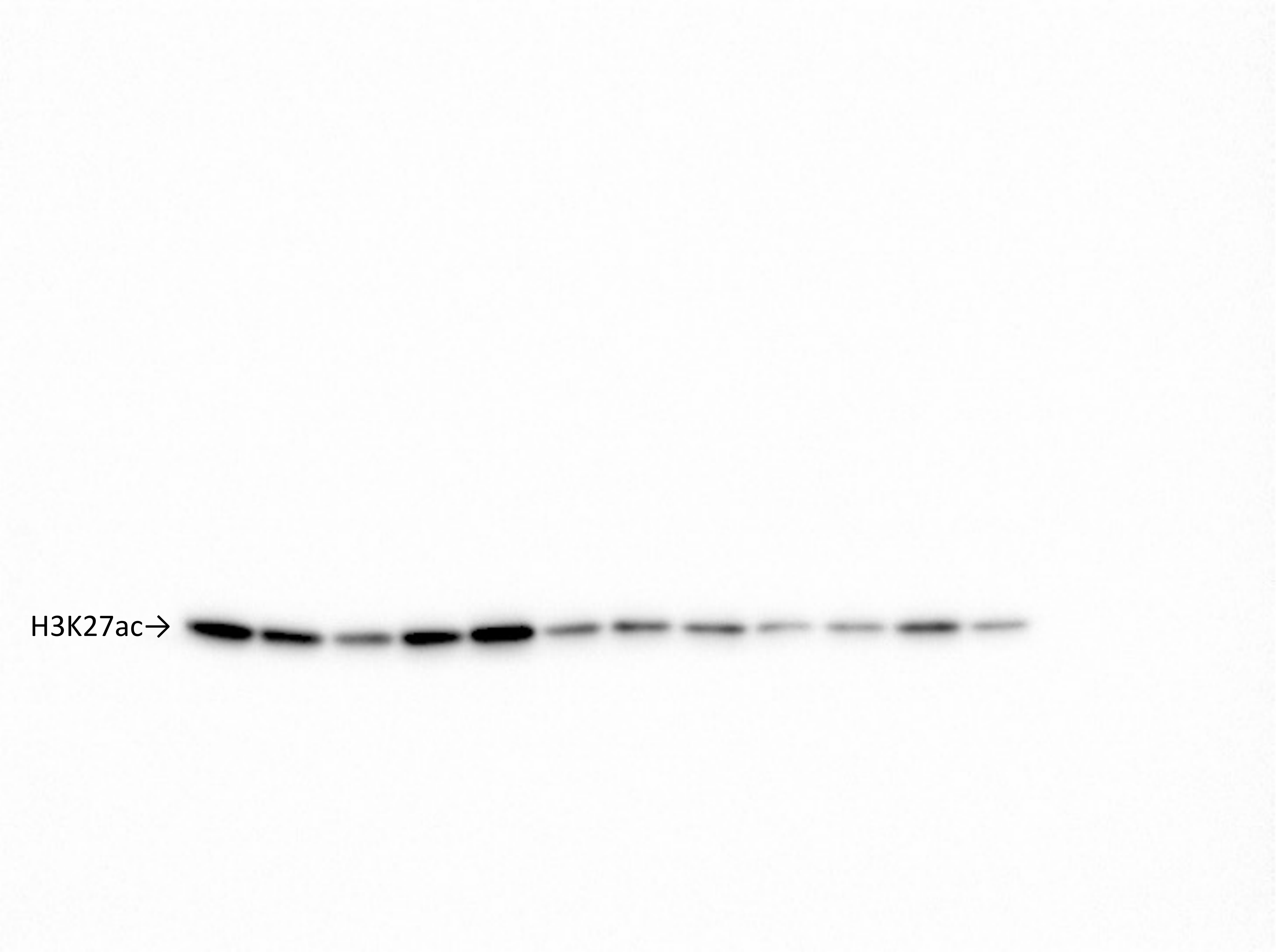

Figure 3A
H3K18ac

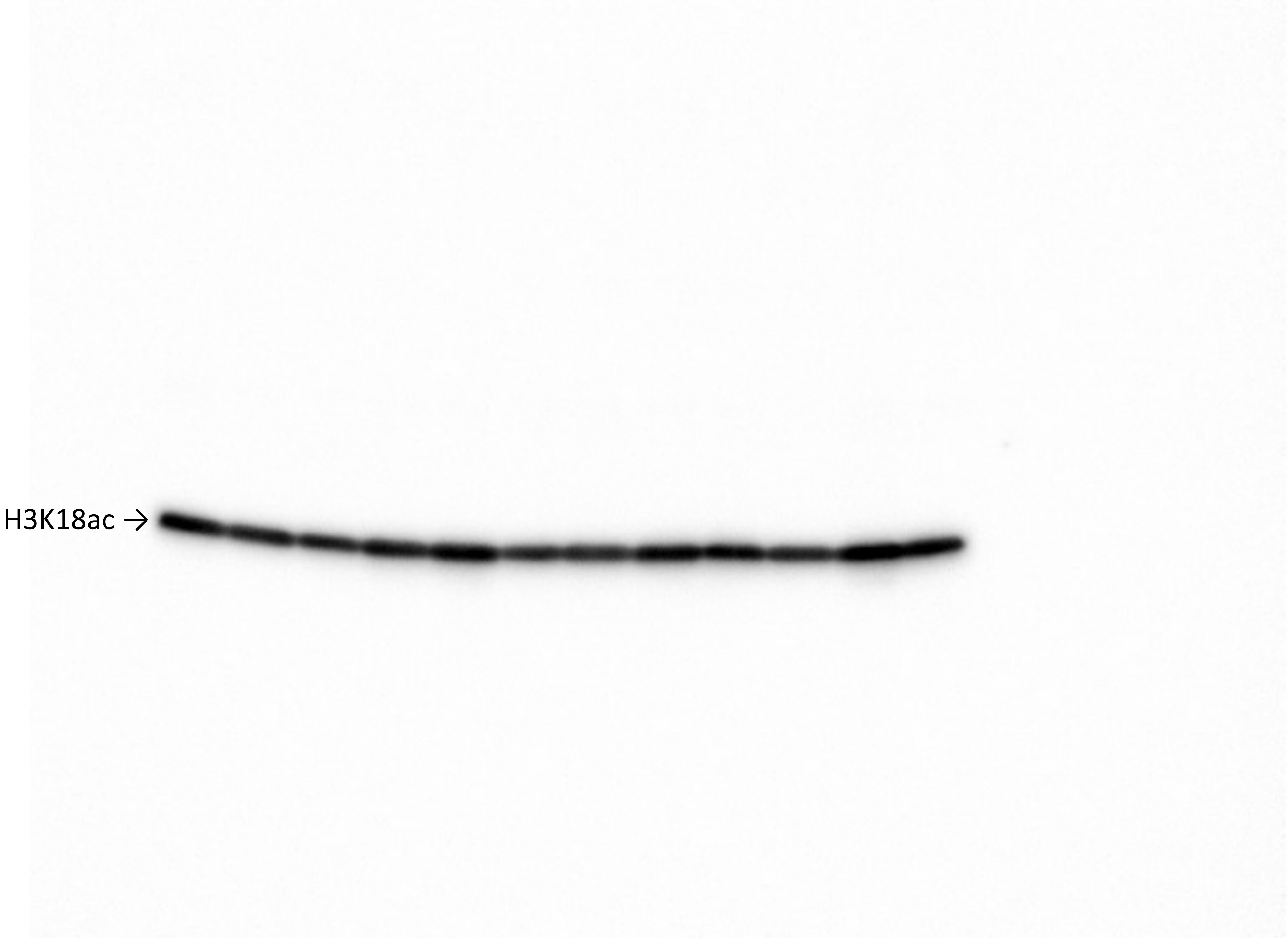

Figure 3A
H3K9me3

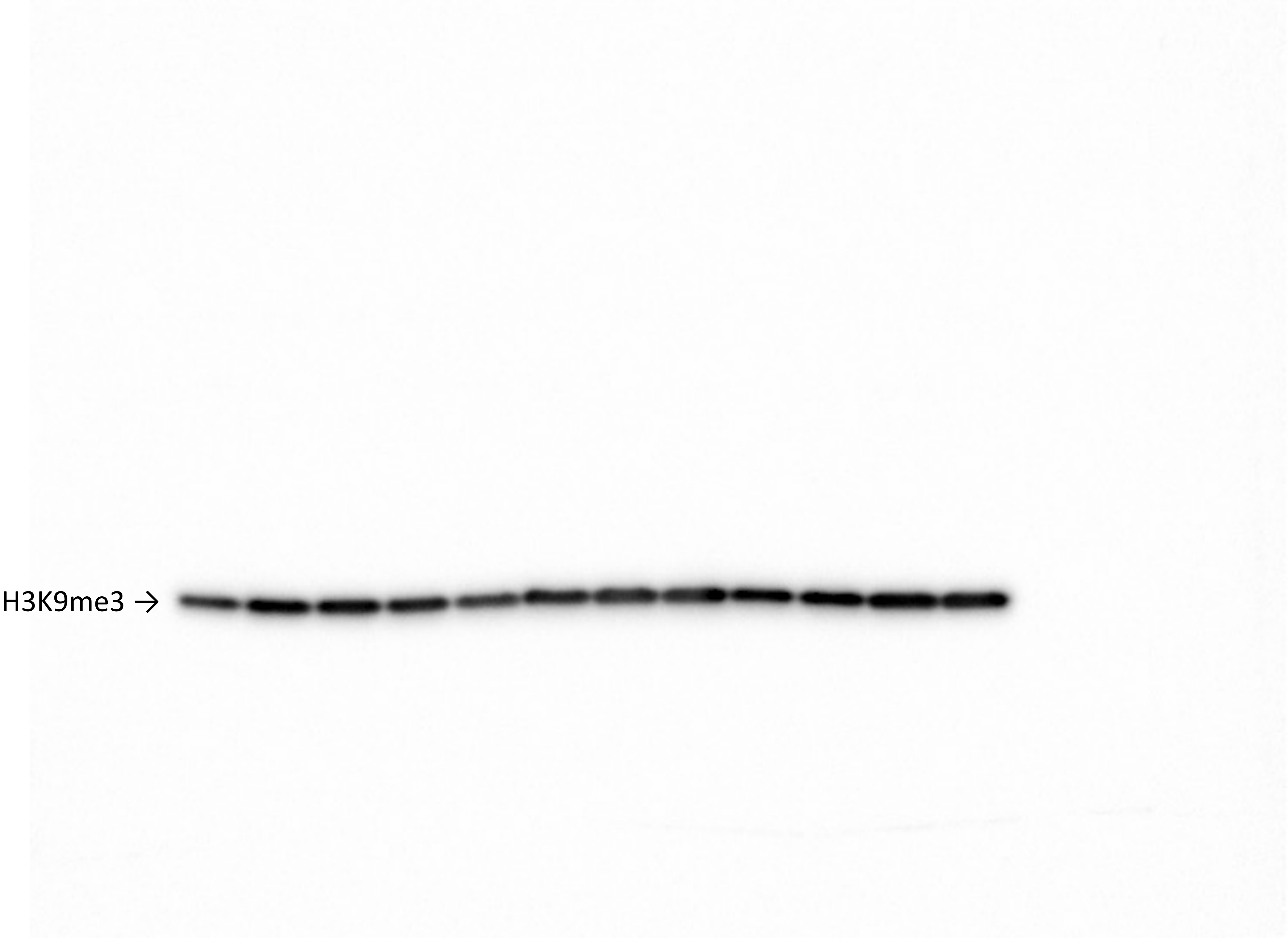

Figure 3A
H3K9ac

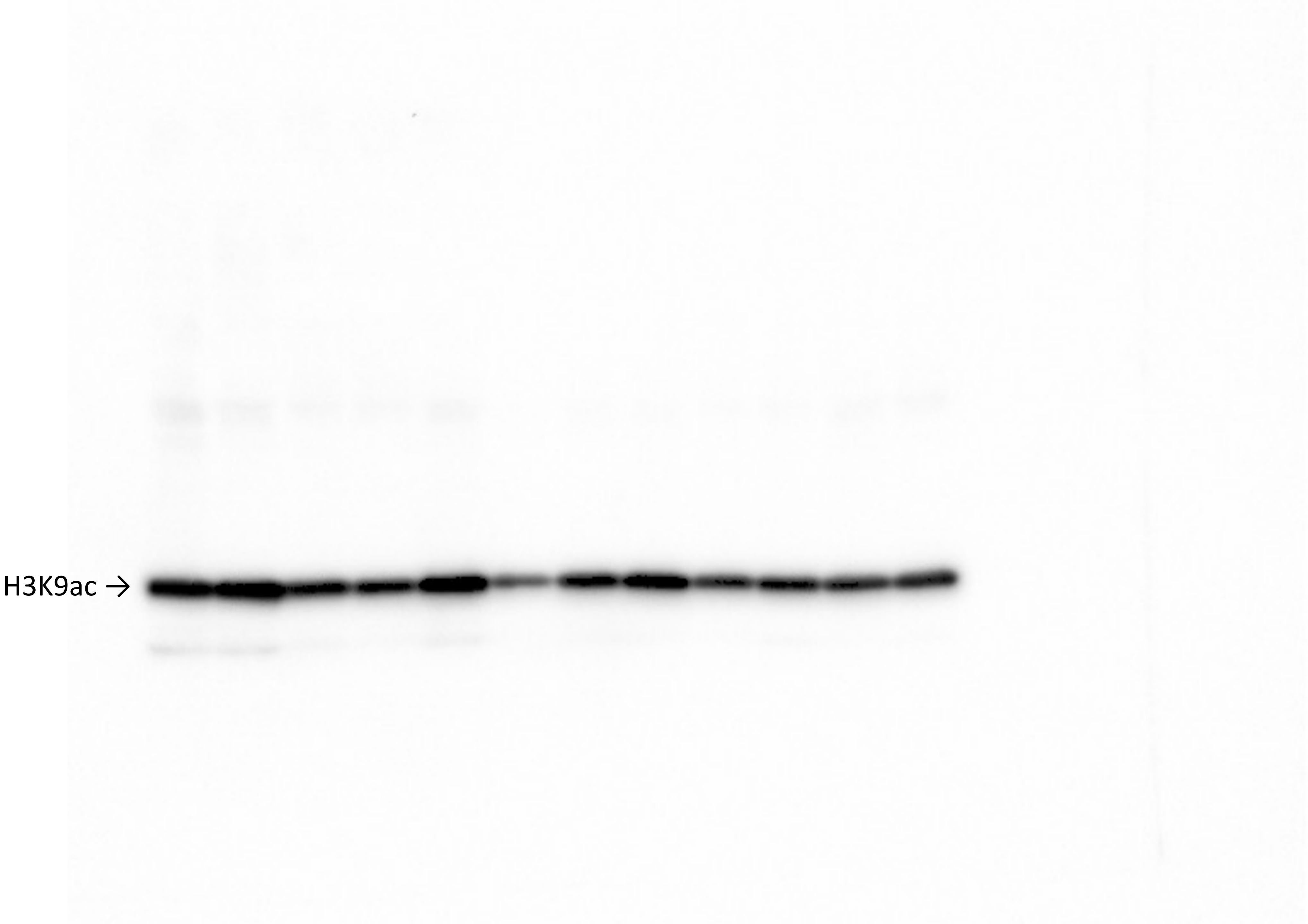

Figure 3A
H3K4me3

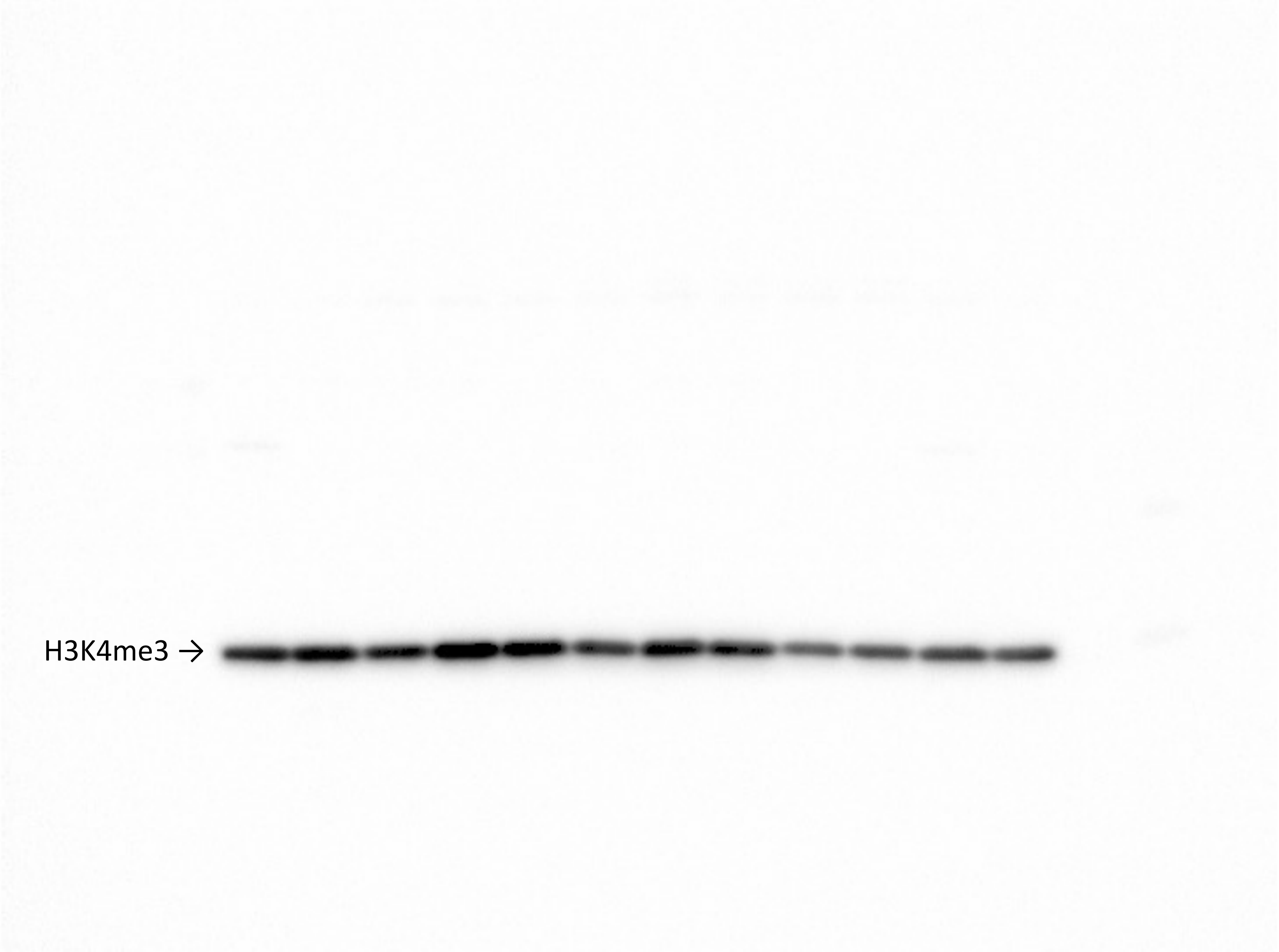

Figure 3A
H3

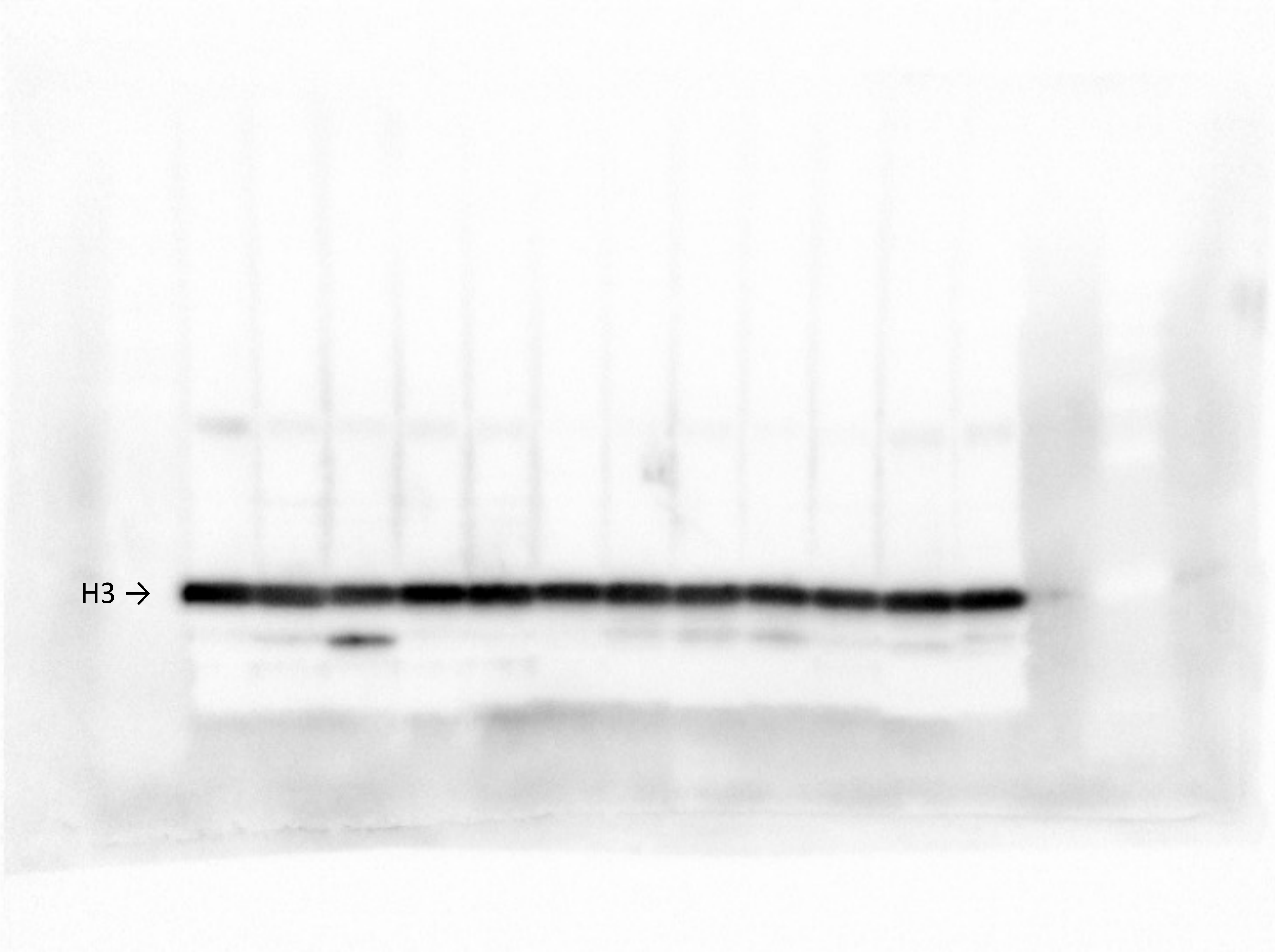

Figure 6C
ITGA3

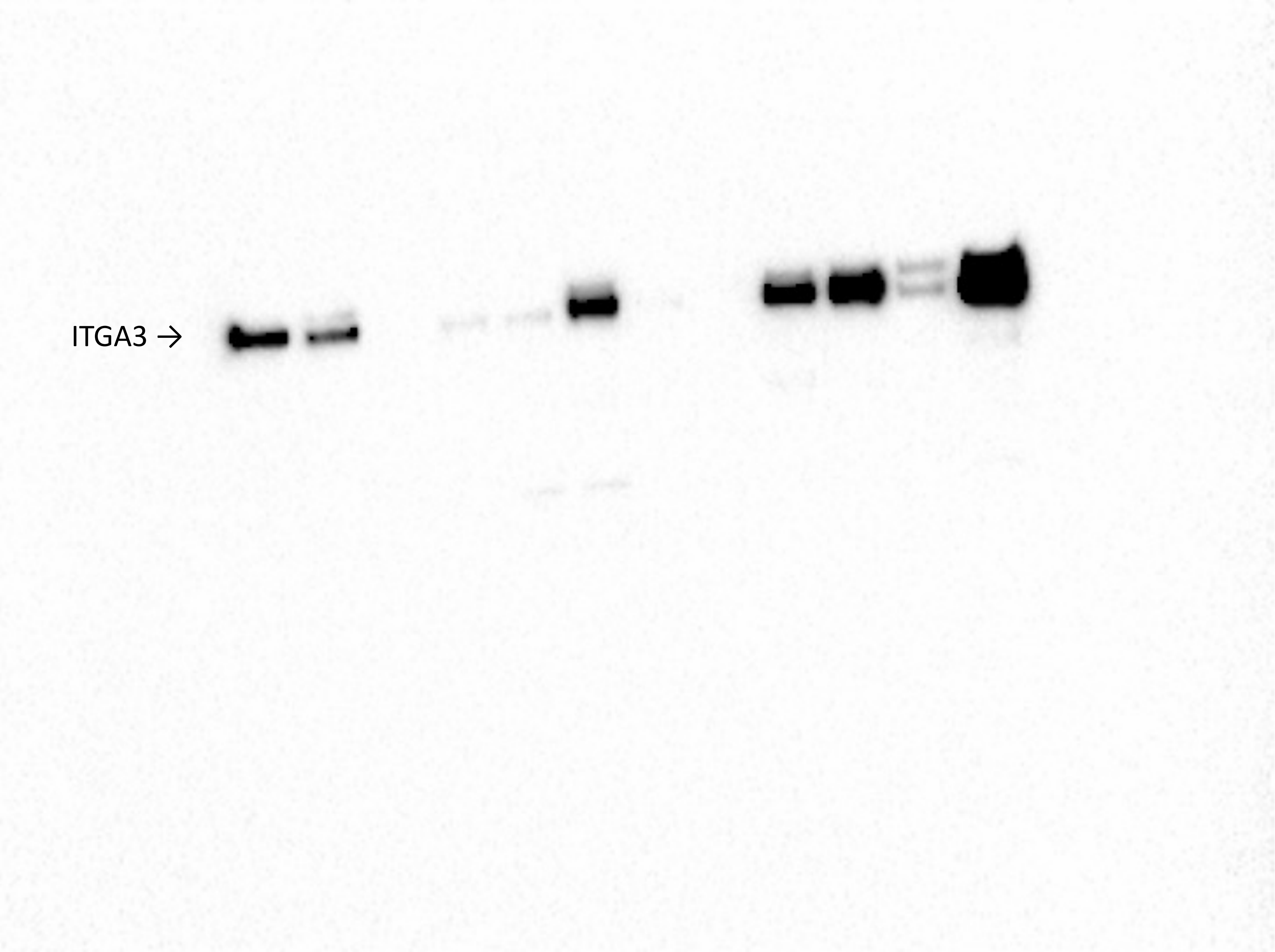

Figure 6C
MITF

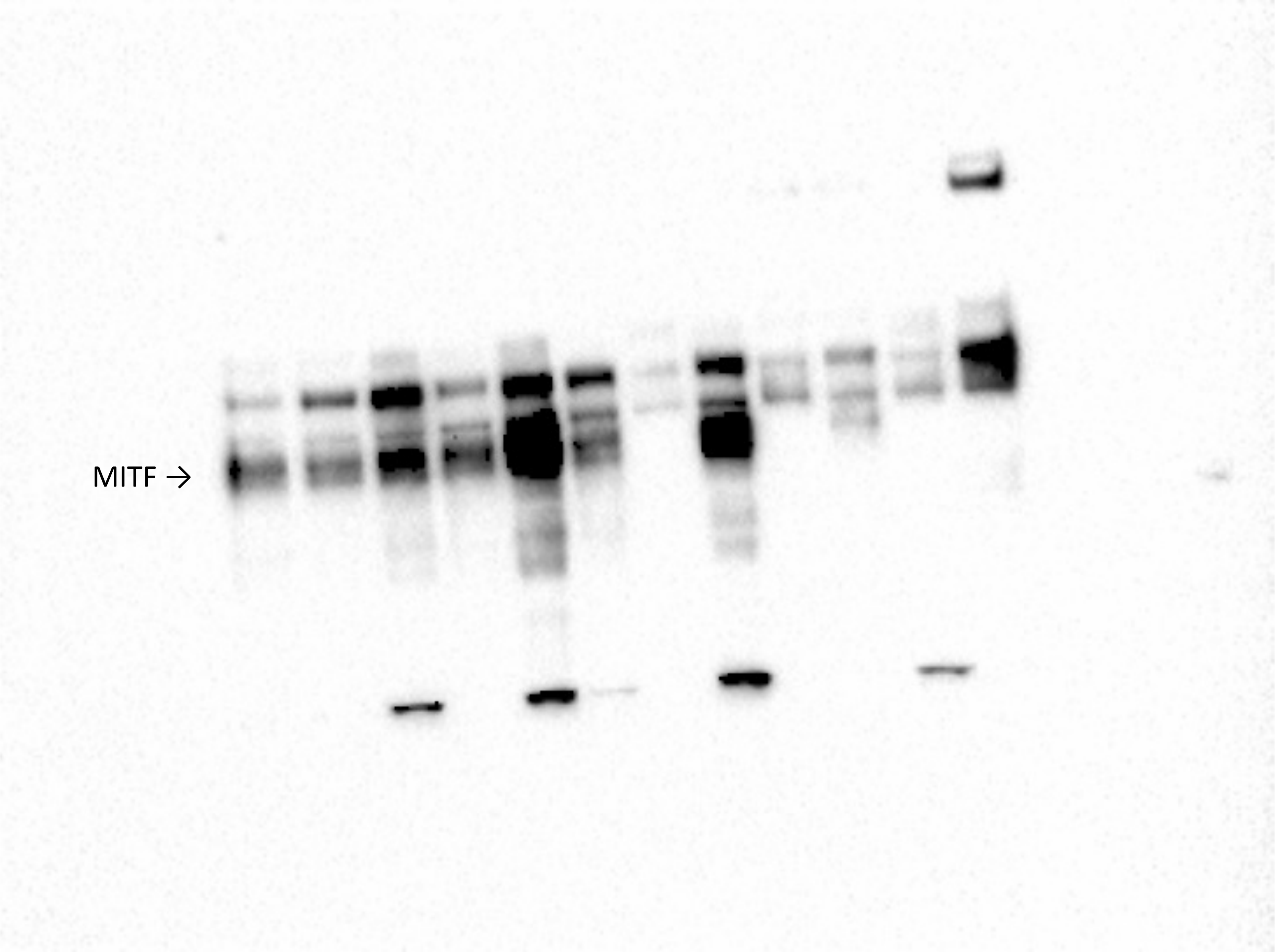

Figure 6C
Beta-actin

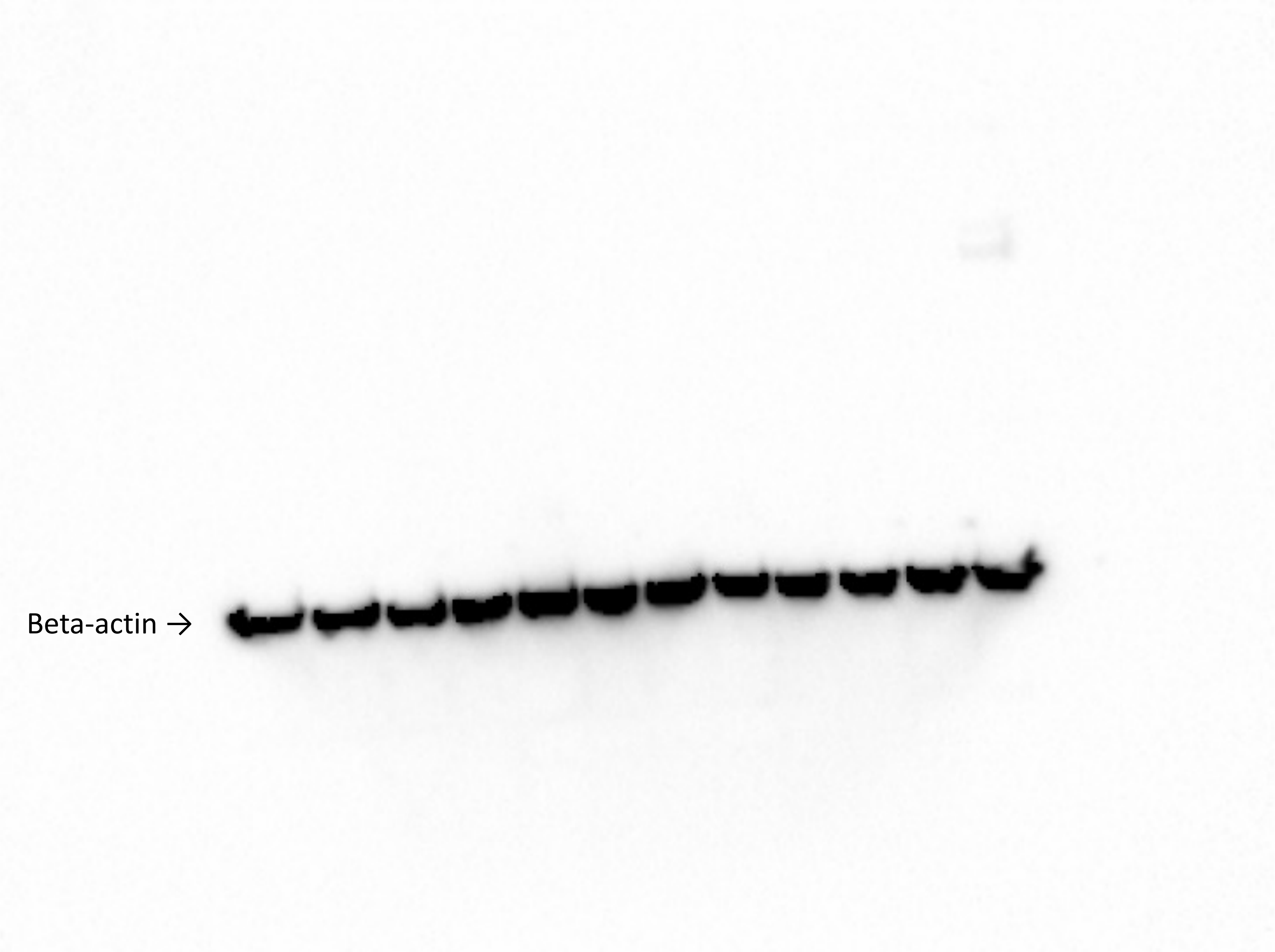

Figure 6D
ITGA3

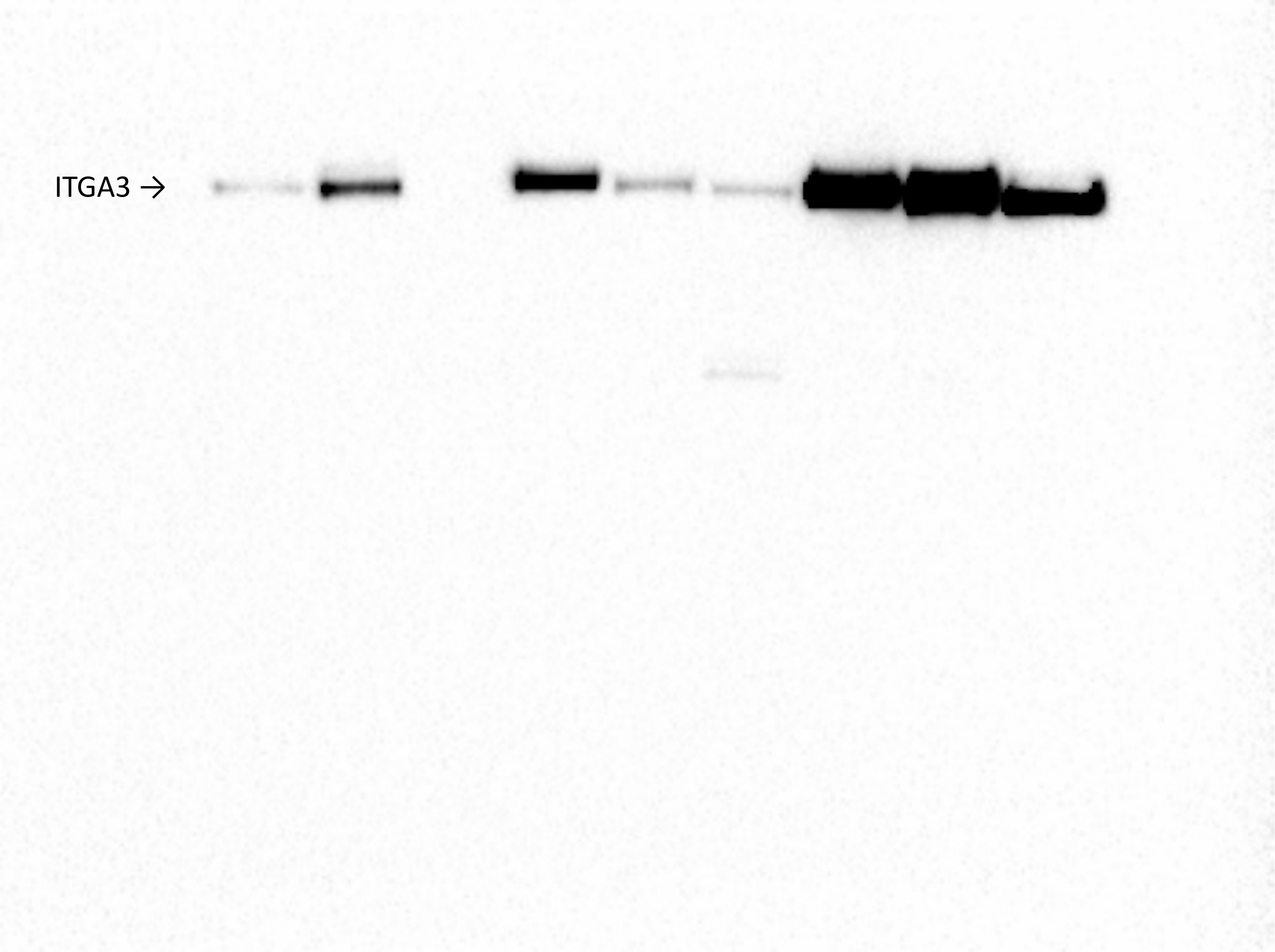

Figure 6D
MITF

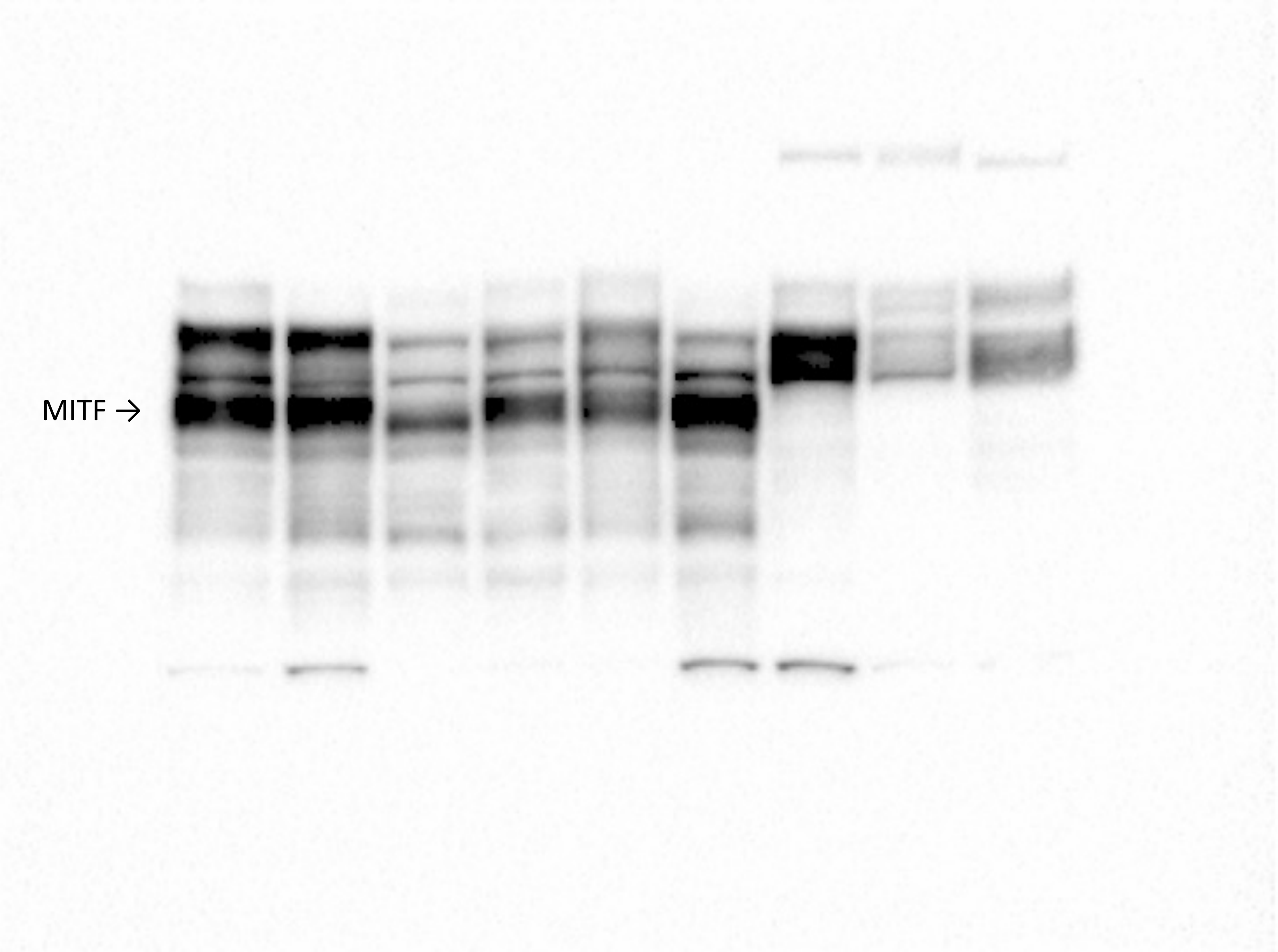

Figure 6D
Beta-actin

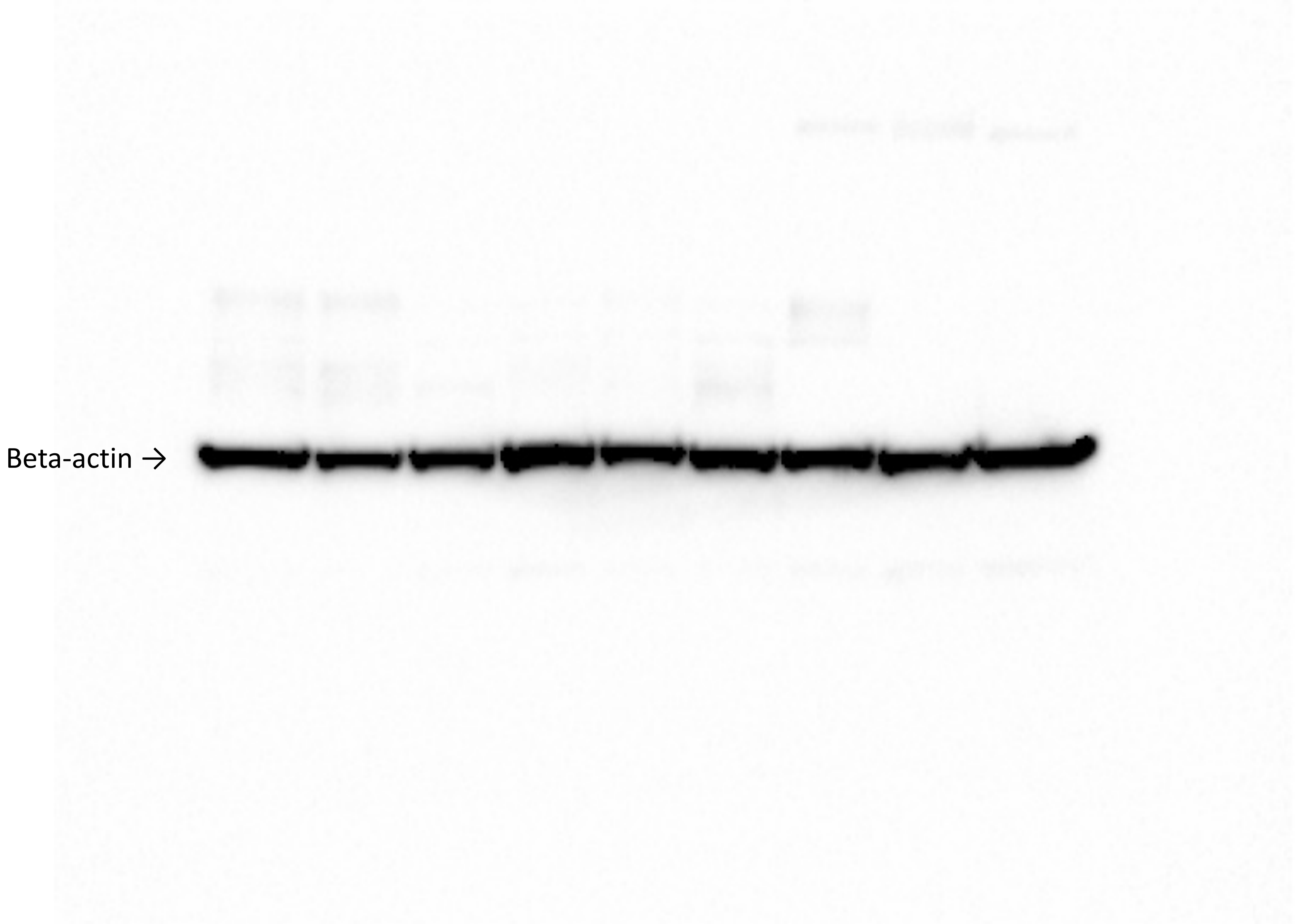

Figure 6E
ITGA3

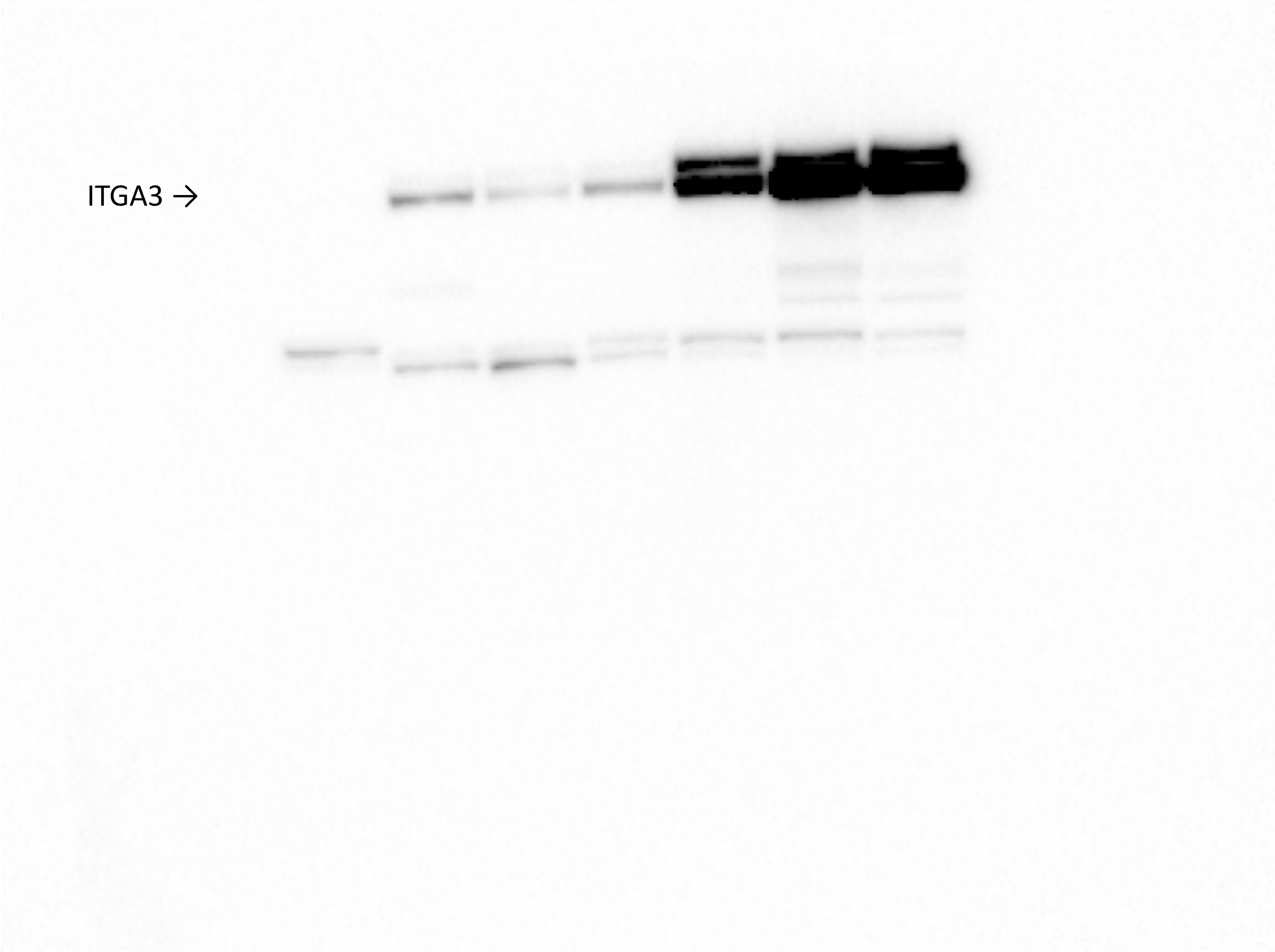

Figure 6E
MITF

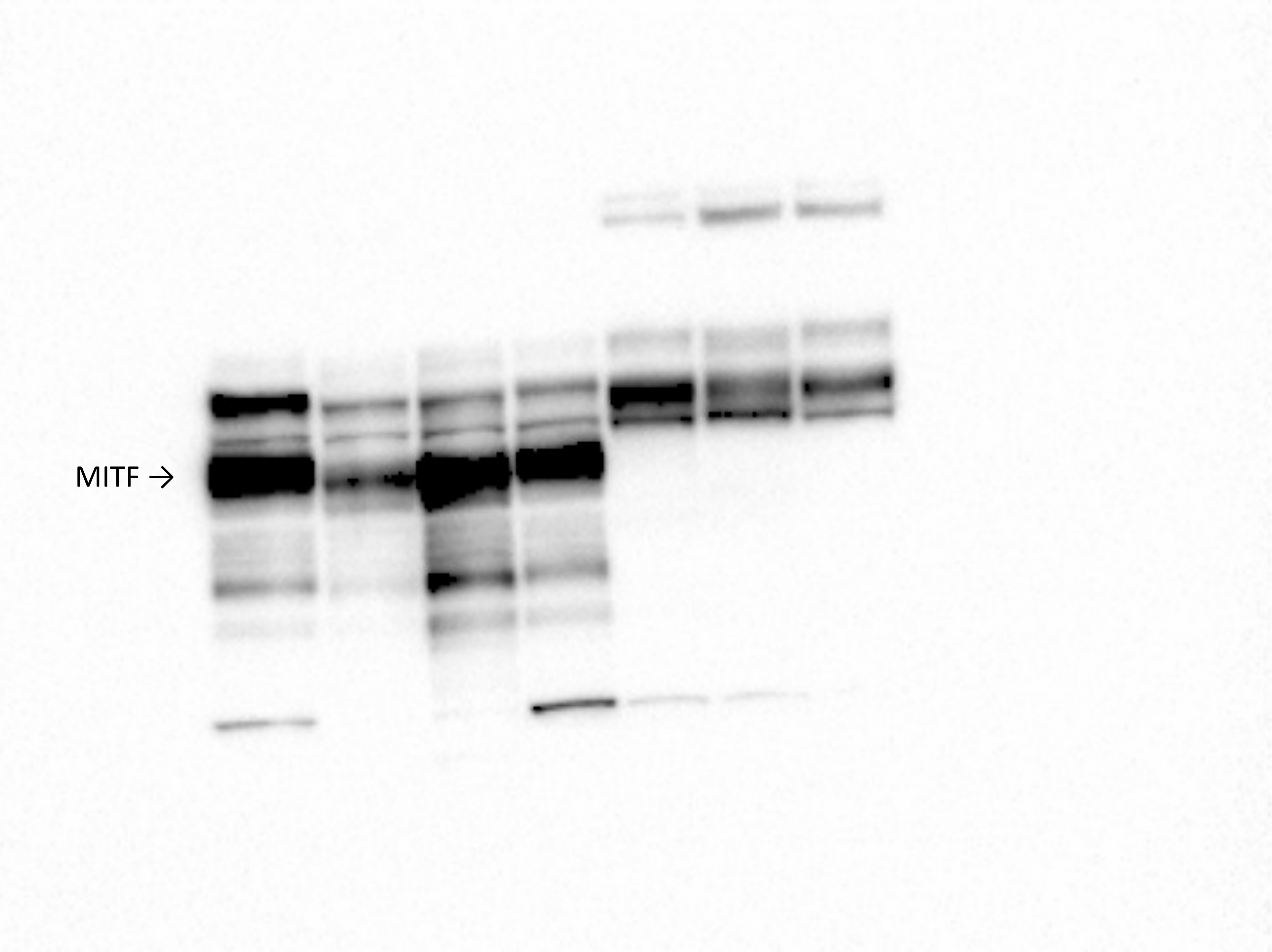

Figure 6E
Beta-actin

Beta-actin →

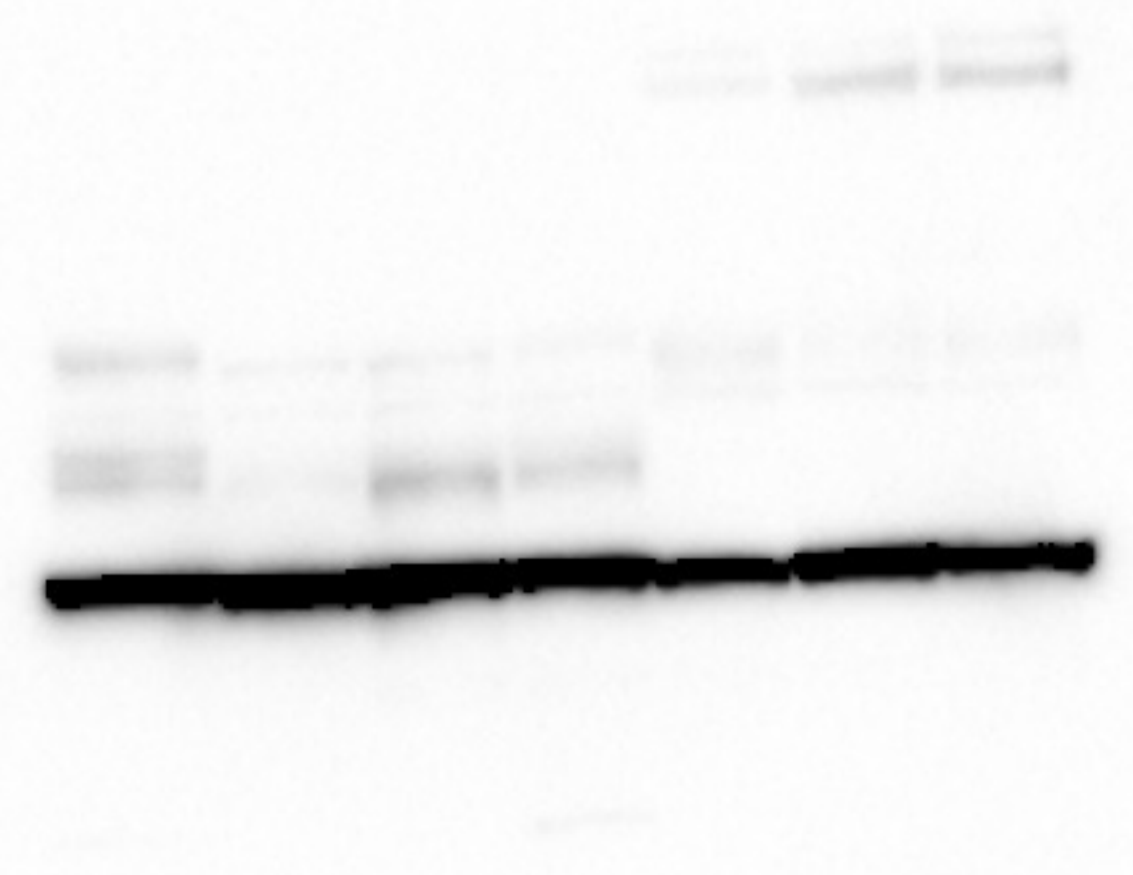

Figure 6F
ITGA3

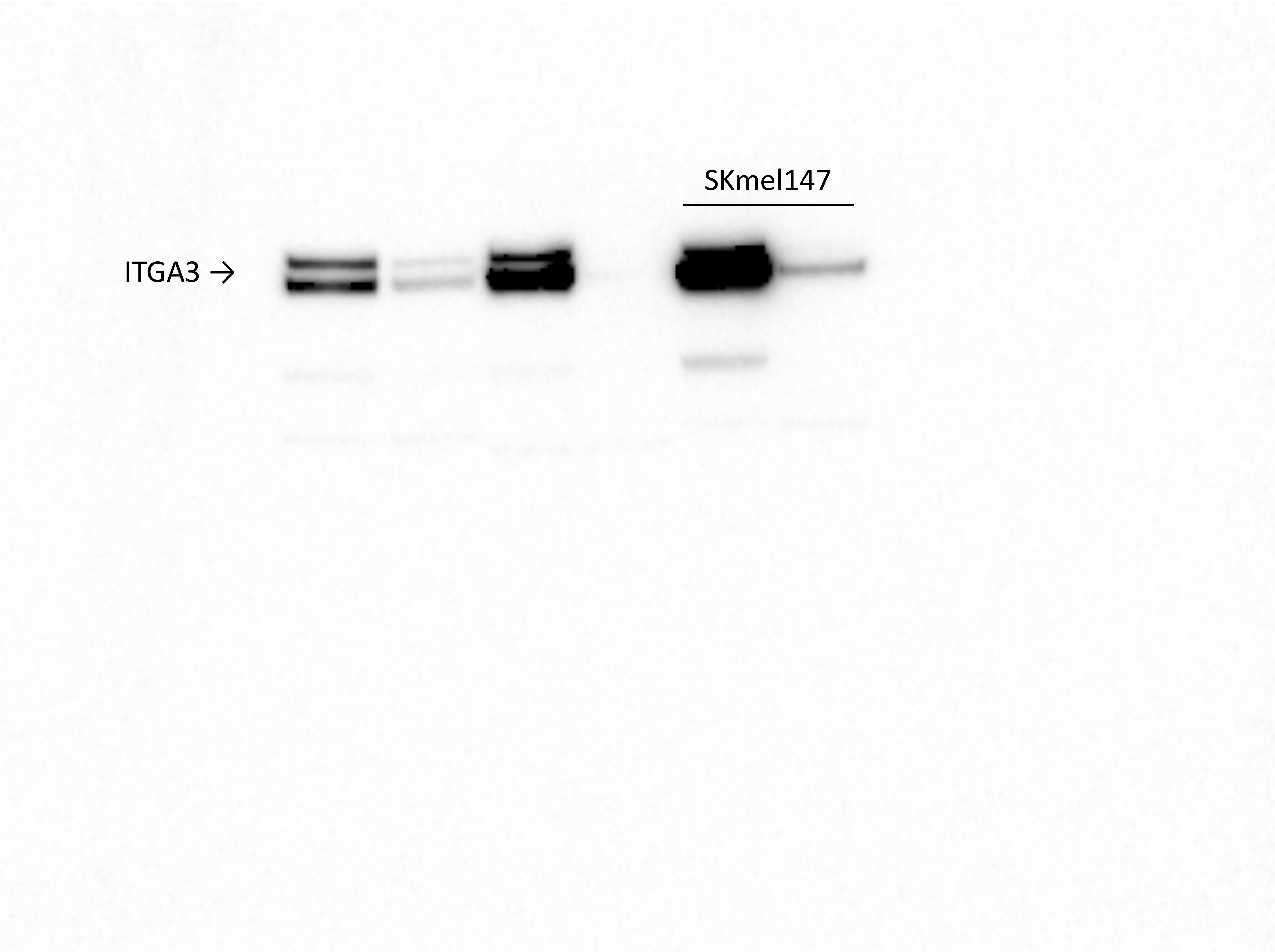

Figure 6F
Beta-actin

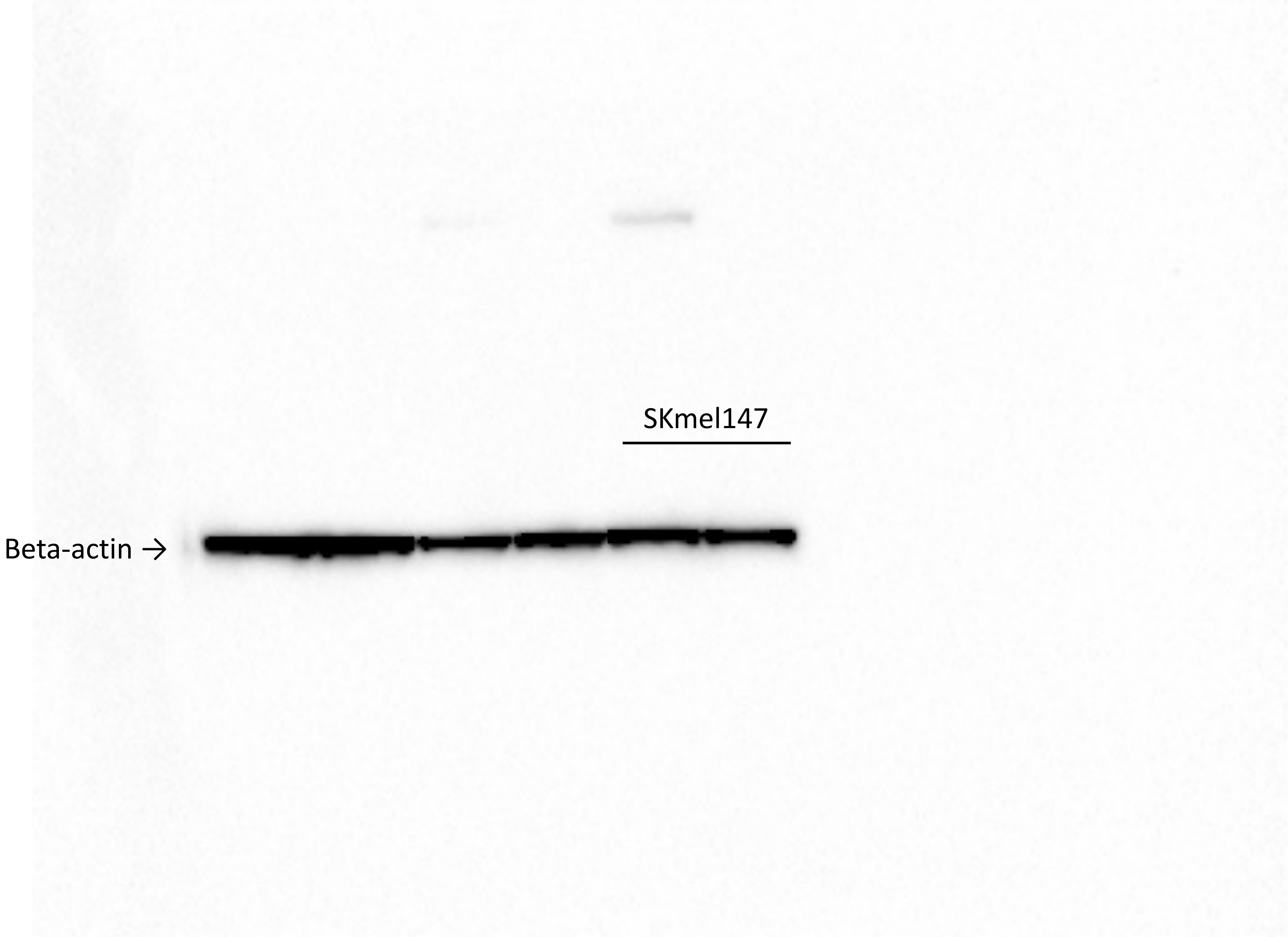

Figure 6G
ITGA3

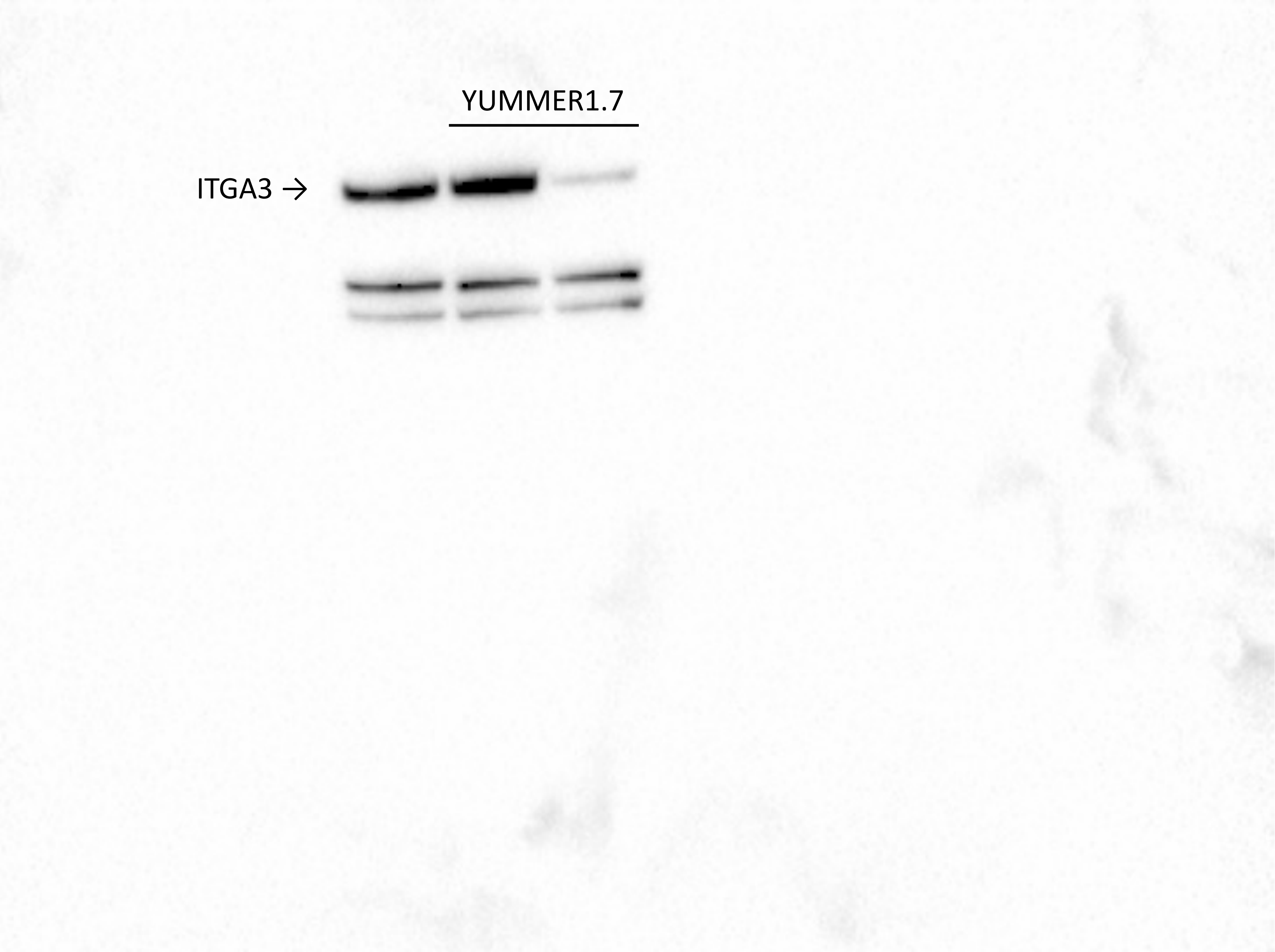

Figure 6G
Beta-actin

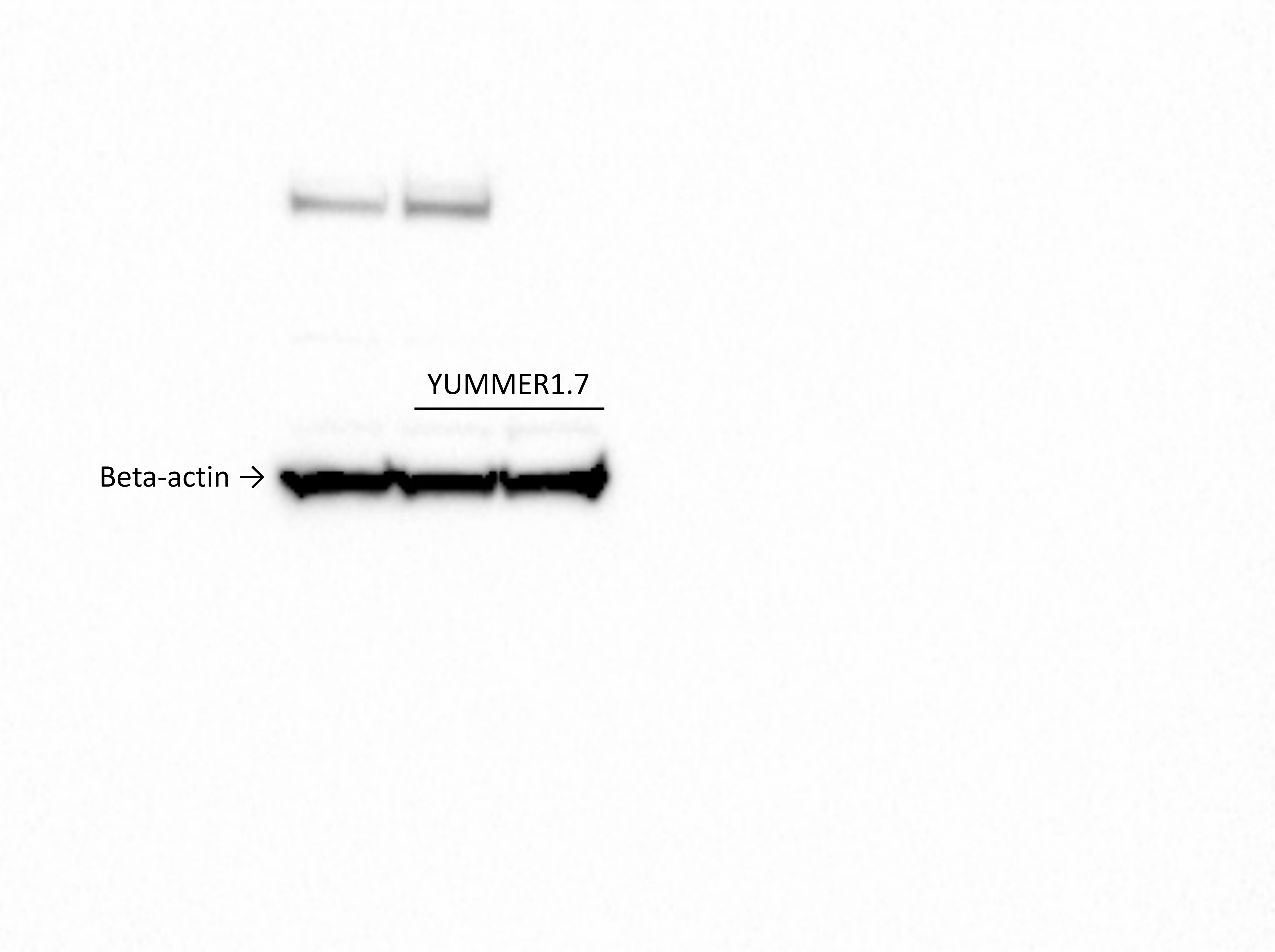

Figure 6H
ITGA3

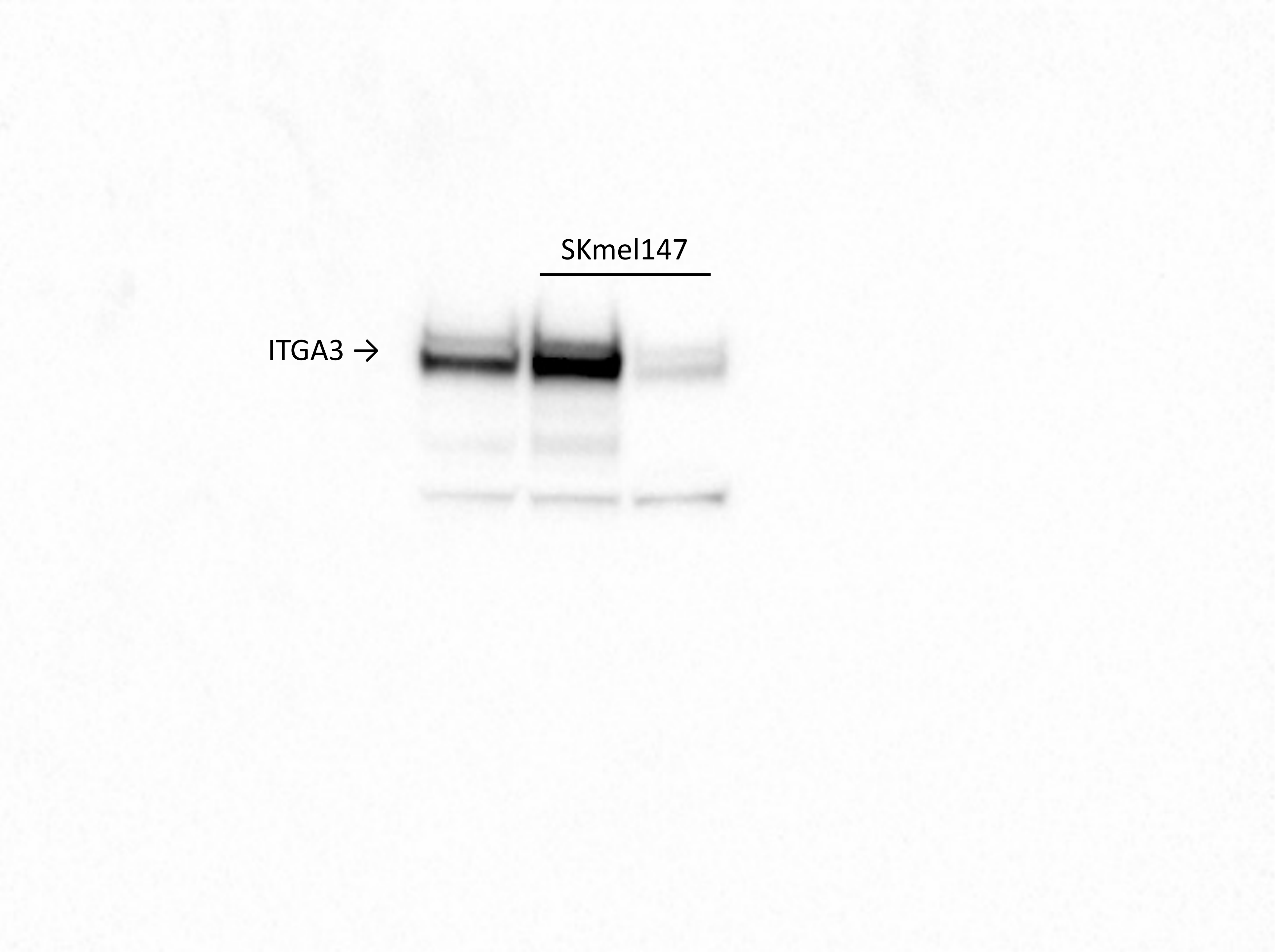

Figure 6H
Beta-actin

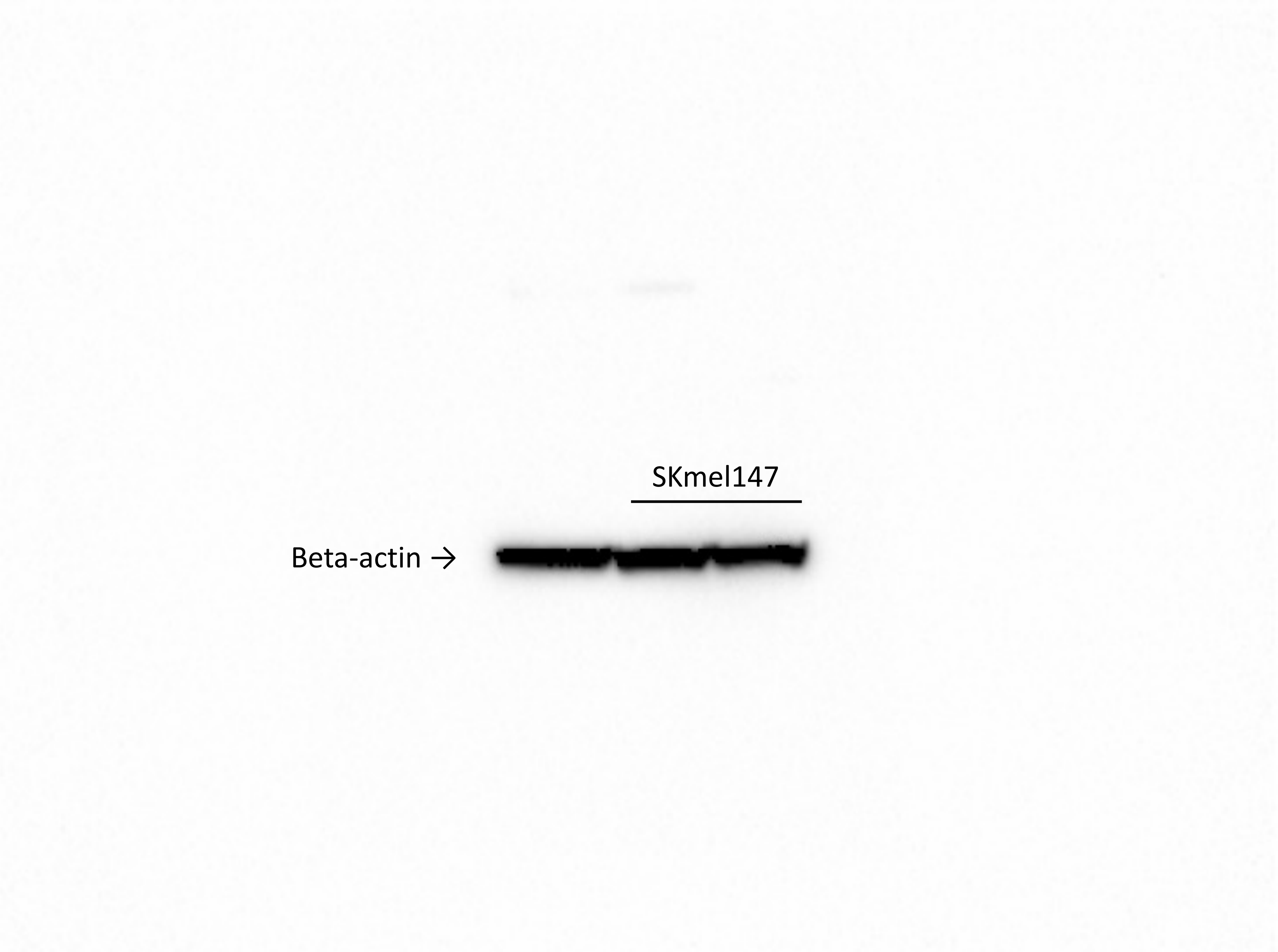

Figure S1C
MITF

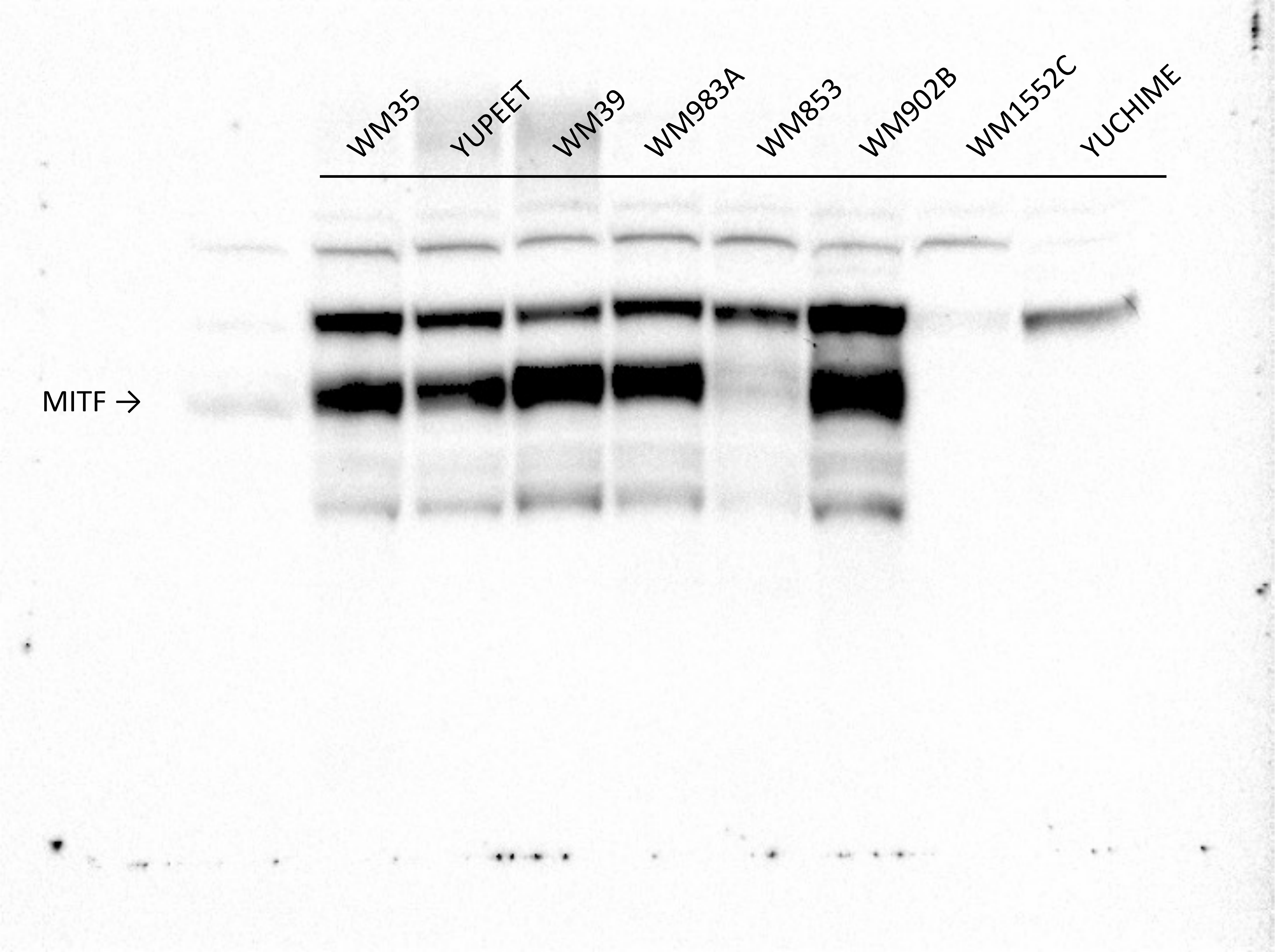

Figure S1C
Beta-actin

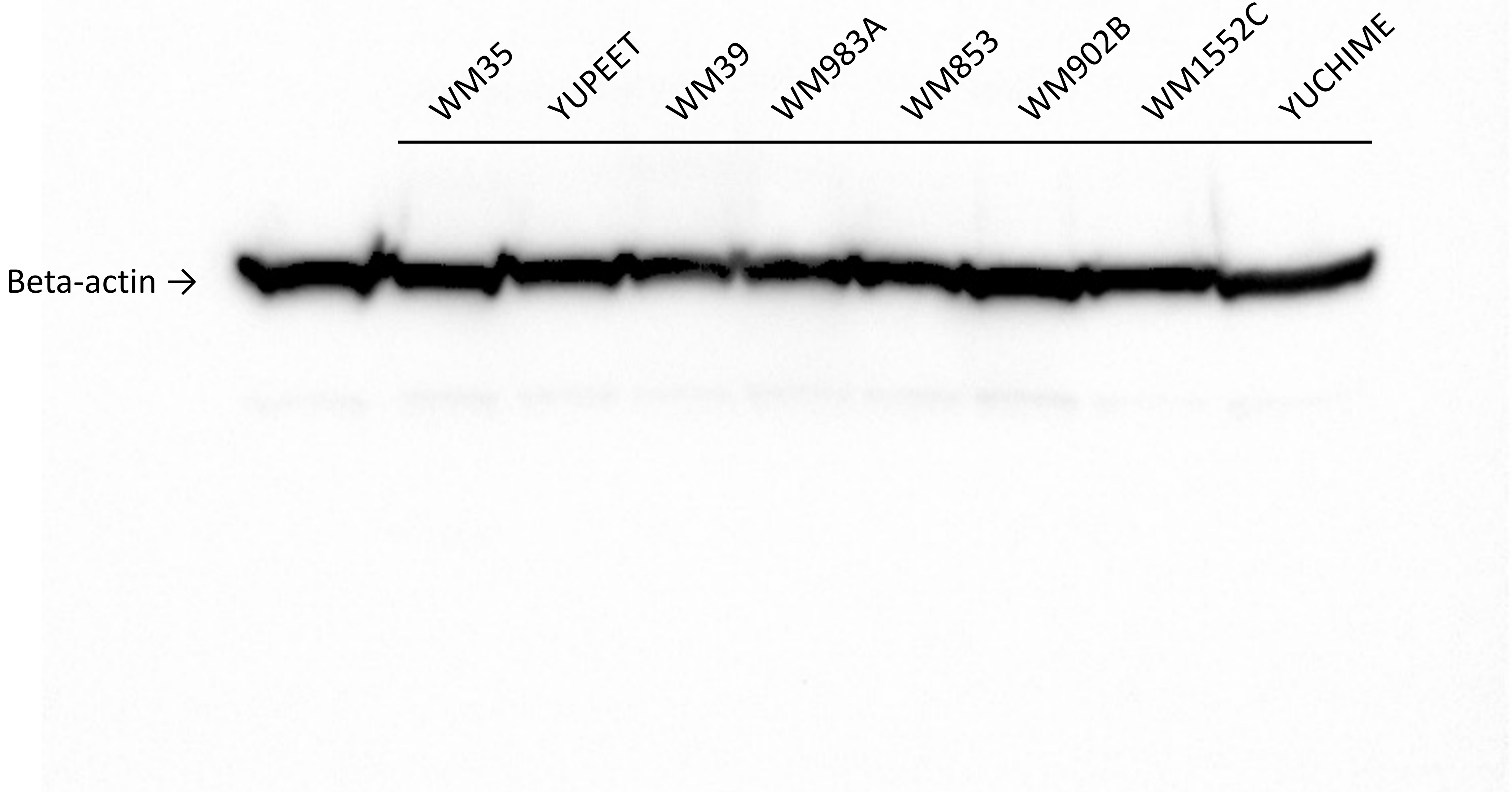

Figure S4
H3K4me1

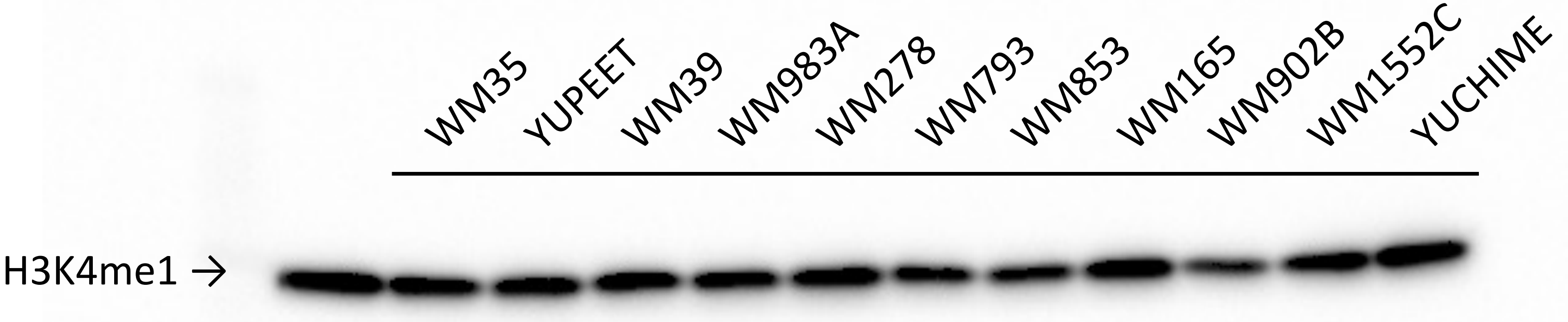

Figure S4
H3K9me2

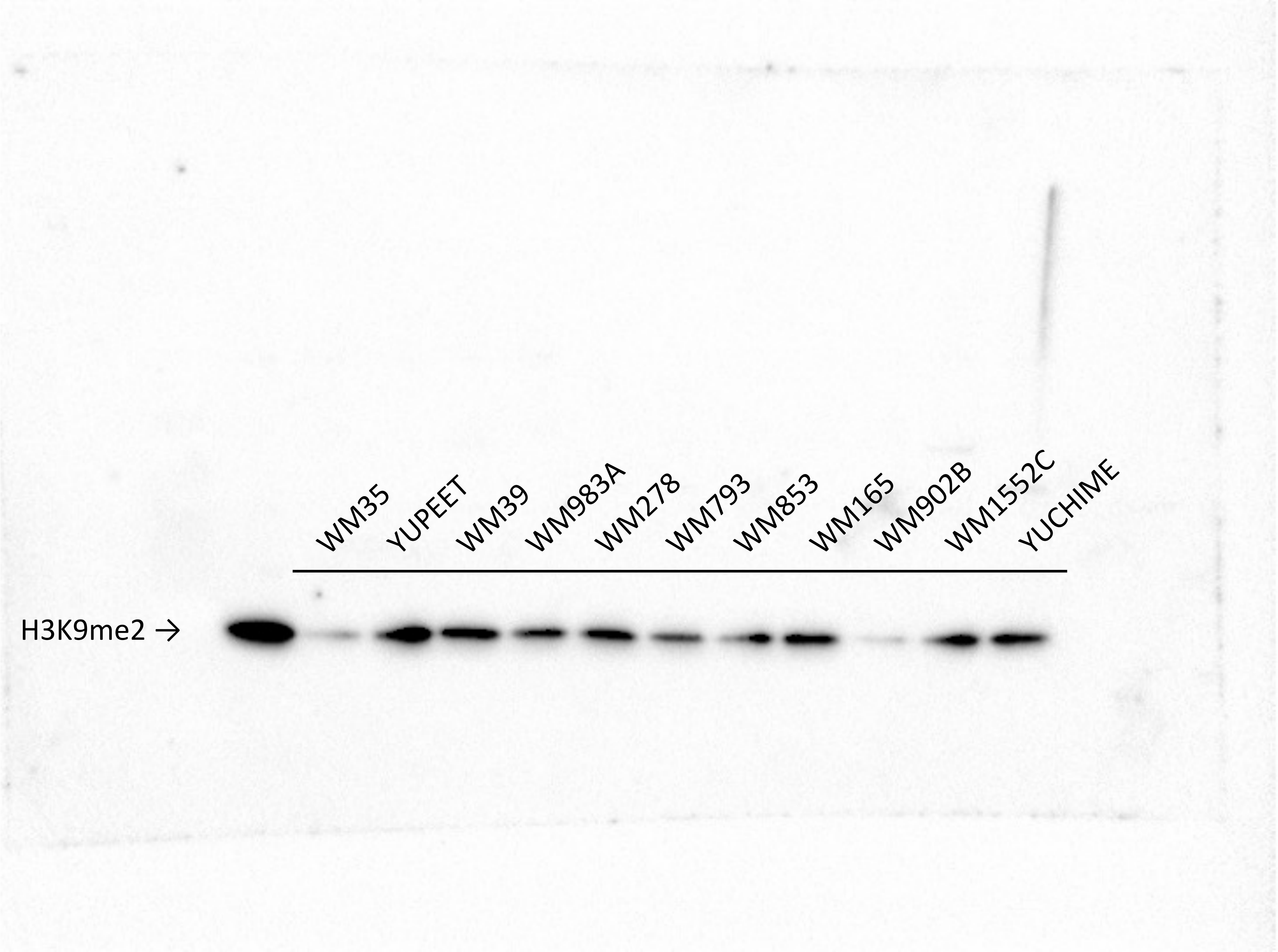

Figure S4
H3K27me3

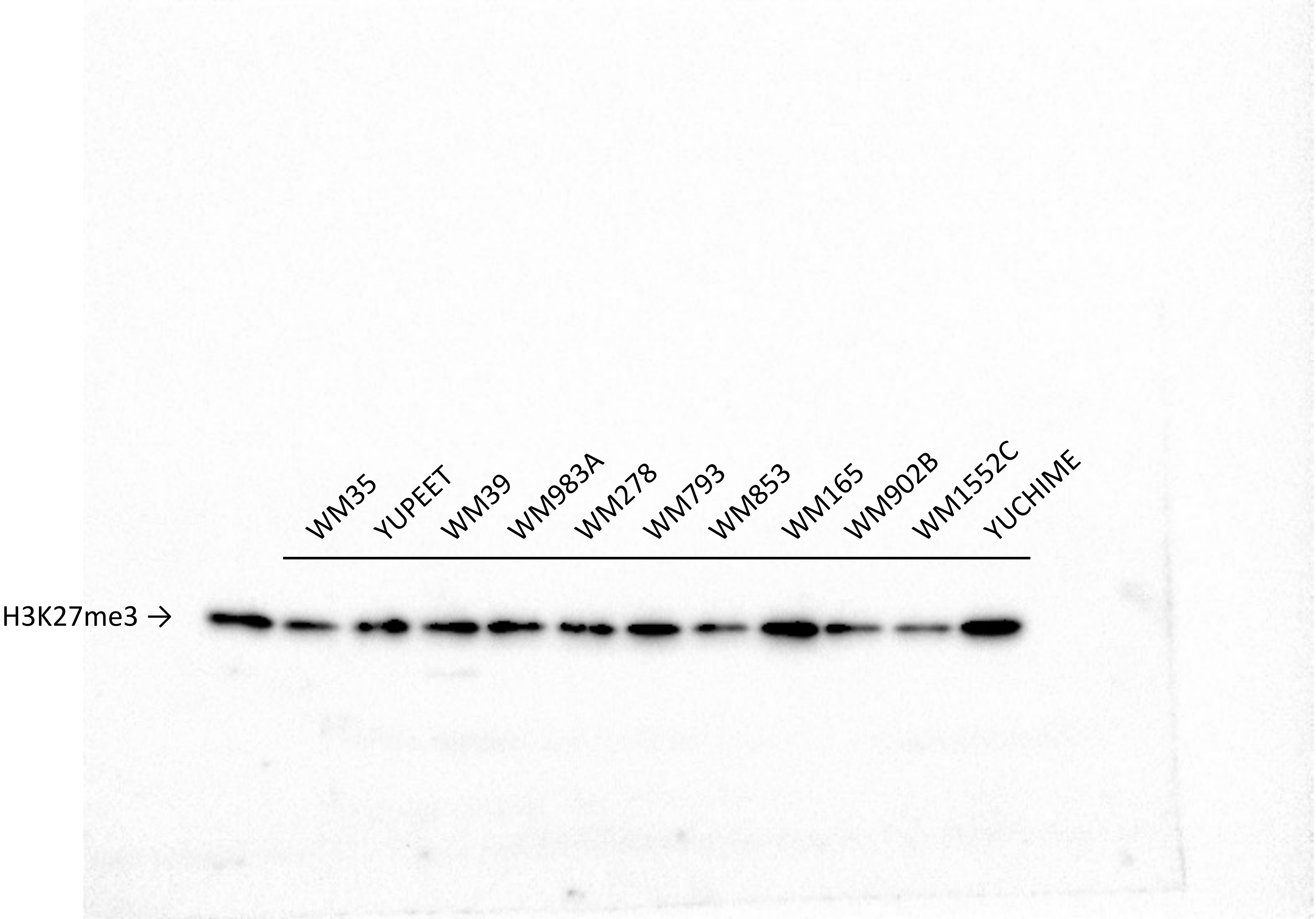

Figure S4
H3K36me3

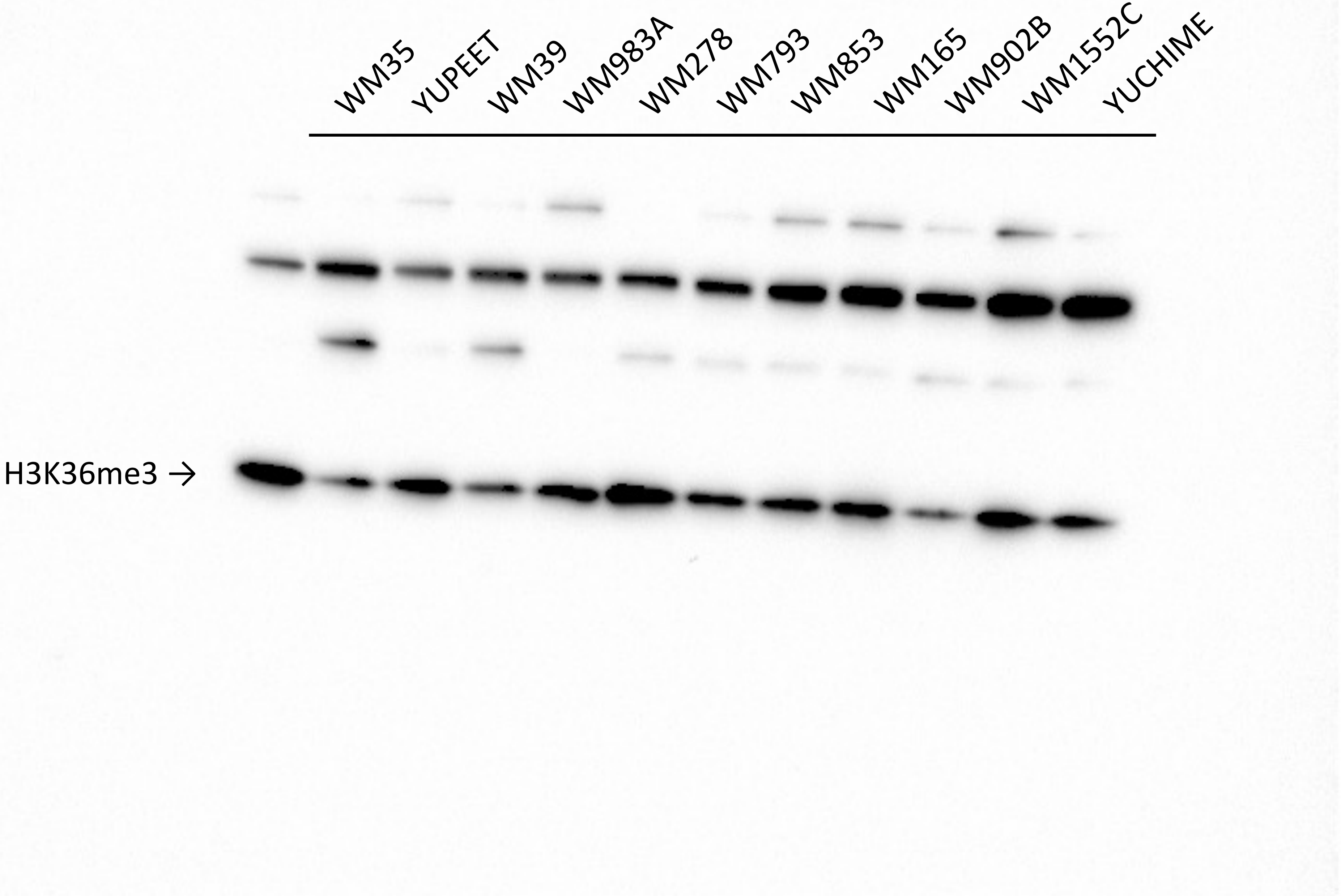

Figure S4
H3

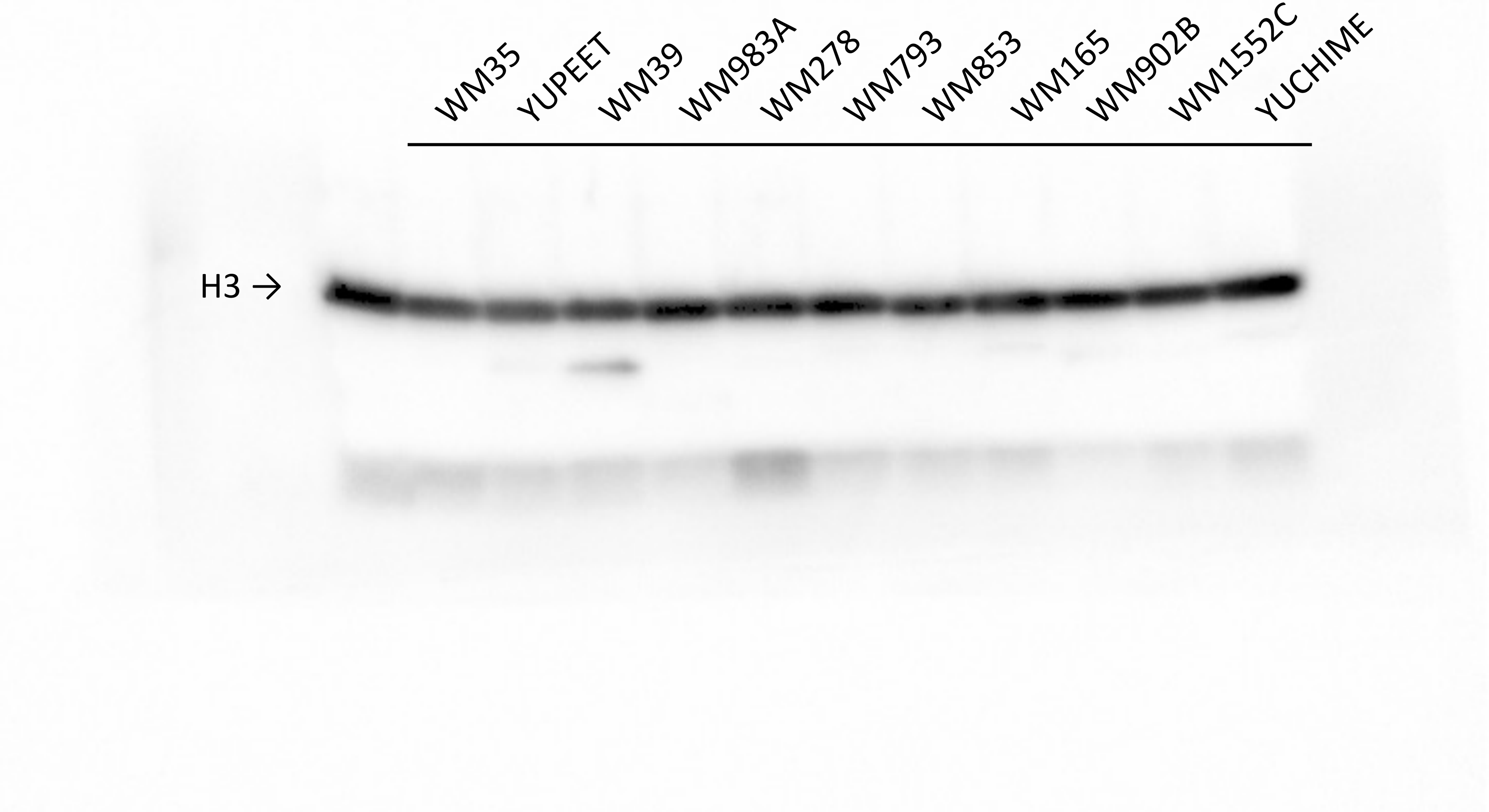
WM35
YUPEET
WM39
WM983A
WM278
WM793
WM853
WM165
WM902B
WM1552C
YUCHIME
H3 →
